# An integrative approach to uncover the components, mechanisms, and functions of traditional Chinese medicine prescriptions on male infertility

**DOI:** 10.3389/fphar.2022.794448

**Published:** 2022-08-11

**Authors:** Xue Bai, Zhejun Liu, Tian Tang, Shujun Yu, Dan Liu, Guimin Liu, Xiaolei Fan, Yibo Tang, Zhenquan Liu

**Affiliations:** ^1^ Beijing Key Laboratory of TCM Basic Research on Prevention and Treatment of Major Disease, Experimental Research Center, China Academy of Chinese Medical Sciences, Beijing, China; ^2^ School of Chinese Materia Medica, Beijing University of Chinese Medicine, Beijing, China; ^3^ School of Traditional Chinese Medicine, Beijing University of Chinese Medicine, Beijing, China

**Keywords:** traditional Chinese medicine, male infertility, mechanism, component, target

## Abstract

Male infertility is a major and growing health problem with an estimated global prevalence of 4.2%. The current therapy is limited by the unknown etiology of MI, emphasizing the critical requirement forward to a more efficient method or medication. Through thousands of years, Traditional Chinese Medicine (TCM) has been shown to be effective in treating MI effectively. However, the components, mechanisms and functions of TCM prescriptions on MI are still obscure, severely limiting its clinical application. In order to discover the molecular mechanism of TCM against MI, our study presents a comprehensive approach integrated data mining, network pharmacology, molecular docking, UHPLC-Q-Orbitrap HRMS, and experimental validation. Here, we begin to acquire 289 clinical TCM prescriptions for MI from a TCM hospital’s outpatient department. Then, Core Chinese Materia Medica (CCMM) was then retrieved from the TCM Inheritance Support System (TCMISS), which was utilized to discover the underlying rules and connections in clinical prescriptions. After that, 98 CCMM components and 816 MI targets were obtained from ten distinct databases. Additionally, the network pharmacology methods, including network construction, GO and KEGG pathway enrichment, PPI analysis, were utilized to reveal that kaempferol, quercetin, isorhamnetin, and beta-sitosterol are the core components of CCMM in treating MI. The mechanisms and functions of CCMM against MI are hormone regulation, anti-apoptosis, anti-oxidant stress, and anti-inflammatory. Furthermore, the strong connections between four core components and six key targets were verified using a molecular docking method. Following that, the core components of the CCMM extract were identified using UHPLC-Q-Orbitrap HRMS analysis. Finally, *in vivo* experiments demonstrated that CCMM and four core components could improve the density, motility, viability of sperm, lecithin corpuscle density, decrease the rate of sperm malformation and testis tissue damage, and regulate the protein expressions of AKT1, MAPK3/1, EGFR, and TNF-α in a mouse model of MI. UHPLC-Q-Orbitrap HRMS analysis and *in vivo* experiments further validated the results of data mining, network pharmacology, and molecular docking. Our study could uncover the components, mechanisms, and functions of TCM prescriptions against MI and develop a new integrative approach to demonstrate TCM’s multi-component, multi-target, and multi-pathway approach to disease treatment.

## 1 Introduction

Male infertility (MI), characterized by impaired sperm function (Agarwal et al., 2019), has become a disease with high incidence, and 75% of these cases are unexplained idiopathic (Okada et al., 2008). Azoospermia, oligozoospermia, asthenospermia, and teratospermia are the four main types of MI (Huynh et al., 2002). Other studies showed that MI is caused by idiopathic blockage, varicocele, immunologic, ejaculatory dysfunction, cryptorchidism, drug/radiation, testicular failure side effects, endocrinology, and other factors (Machen and Sandlow, 2020). Furthermore, a recent study found that testicular-borne may have an impact on sperm quality (Kiyozumi et al., 2020; Lord and Oatley, 2020). Assisted reproductive technology (ART), which has effectively improved the birth rate of infertile couples, is currently the most prevalent treatment for treating MI (Qin et al., 2015). But, there are still unsolved ART issues, such as high cost, possible safety risks, and treatment unpredictability. Moreover, ART did not significantly enhance the sperm quality of MI patients (Rhoton-Vlasak et al., 2020). Moreover, Coronavirus Disease 2019 (COVID-19) has been recognized as being caused by SARS-CoV-2 (Romagnoli et al., 2020). Some investigations have indicated that SARS-CoV-2 can harm testicular functioning directly or indirectly through secondary inflammatory and immunological reactions, eventually leading to MI (Xu et al., 2006; Aitken, 2020; Illiano et al., 2020; Segars et al., 2020; Singh et al., 2020; Wang et al., 2020; Youssef and Abdelhak, 2020). As a result, more effective therapy or medication to treat MI is urgently needed.

TCM has been shown to cure various diseases, such as MI, for thousands of years (Zhou et al., 2019). According to TCM theory’s concept of “ZHENG” and syndrome differentiation (Li et al., 2007; Li et al., 2014), TCM treatment is characterized by the formulation of different clinical TCM prescriptions in response to the patients’ constitutional indications and symptoms. But, revealing the underlying laws and rules of a large number of TCM prescriptions on MI is still an unsolved problem. Besides, the components, mechanisms, and functions of TCM prescriptions in treating disease remain unclear.

Data mining is a computational method that automates the extraction of information from large amounts of data to discover innovative insights (Hand, 2007). Recent studies have shown TCM’s conception of holism is similar to the theoretical principles of system pharmacology, that the conventional “one target, one drug” strategy transformed with a novel “network target, multi-component” approach (Hopkins, 2008; Shao and Zhang, 2013). Molecular docking, a technique for predicting binding sites, determines the relationships between ingredients and targets (Ping et al., 2019). Ultra high-performance liquid chromatography-Q Exactive hybrid quadrupole-orbitrap high-resolution accurate mass spectrometry (UHPLC-Q-Orbitrap HRMS), which is able to accurately determining the mass of unknown substances, has developed into a critical instrument for identifying chemical components in natural products (Wang et al., 2016; Sun et al., 2017). Studies showed that cyclophosphamide (CP) could impair reproductive functions in males, leading to spermatogenesis disorder and male infertility (Elangovan et al., 2006; Çeribaşi et al., 2010).

Therefore, in this study, we adopted a comprehensive approach integrated data mining, network pharmacology, molecular docking, UHPLC-Q-Orbitrap HRMS, and *in vivo* experimental validation. Firstly, the clinical TCM prescriptions in treating MI came from the outpatient department of a TCM hospital. Secondly, TCM Inheritance Support System (TCMISS) was used for uncovering the underlying rules and connections in clinical TCM prescription and retrieving Core Chinese Materia Medica (CCMM). Thirdly, various databases were conducted to collect the components and targets of CCMM and MI. Then, we performed the network pharmacology methods, such as network construction, GO and KEGG pathway enrichment, PPI analysis, to explore the core components, key targets, and molecular mechanisms of CCMM in TCM prescriptions on MI. After that, the interactions between the core components and key targets were explored utilizing molecular docking. Next, the core components in CCMM of TCM prescriptions against MI were identified by UHPLC-Q-Orbitrap HRMS. Finally, a mouse model of MI induced by CP was established to research the components, mechanisms, and functions of CCMM in TCM prescriptions in treating MI, and further estimate the results of data mining, network pharmacology, and molecular docking.

## 2 Materials and methods

### 2.1 Data mining

#### 2.1.1 Prescriptions collection

The outpatient department of a TCM hospital affiliated to Beijing University of Chinese Medicine provided the TCM prescriptions used to treat MI. The following inclusion criteria apply to TCM prescriptions: (1) the patient was initially diagnosed with MI, which includes azoospermia, oligozoospermia, asthenospermia, and teratospermia; (2) the patient was over the age of 23; (3) the patient has been married for more than one year and had normal sex without contraception for 12 months, but the woman was unable to conceive due to male factors; (4) There is no family history of MI in the patient’s family. The exclusive criterion is that the patient’s wife suffers from an illness that makes conception difficult.

#### 2.1.2 Core Chinese materia medica extraction from clinical traditional Chinese medicine prescriptions

TCMISS software (V2.5) comprises six functional modules: clinical collection, platform management, data management, knowledge retrieval, statistical report generation, and data analysis. Three graduate students were in charge of the clinical TCM prescriptions gathering process. One of them utilized the “clinical collection” feature to gather prescriptions, while the others checked the data using the “platform management” function. After analyzing the frequency of Chinese materia medica (CMM), the combinations of CCMM were obtained using the “data analysis” function. The principle of screening CCMM by using TCMISS software is association rules analysis and complex system entropy methods. The degree of support was 140, and the level of confidence was more than or equal to 0.95.

### 2.2 Network pharmacology

#### 2.2.1 Components of core Chinese materia medica

Two TCM databases, TCMID (http://119.3.41.228:8000/tcmid/) (Huang et al., 2018) and TCMSP (http://tcmspw.com/tcmsp.php) (Ru et al., 2014) (Supplement 1) were used to acquire the components of CCMM. Then, a Venn diagram (Bardou et al., 2014) and two ADME-related models (Walters and Murcko, 2002; Xu et al., 2012), drug-likeness (DL) ≥ 0.18 and oral bioavailability (OB) ≥ 30% (Feng et al., 2018), were used to search for bioactive components. Following that, we examined PubChem (https://pubchem.ncbi.nlm.nih.gov/) (Kim et al., 2019) and ALOGPS2.1 (http://www.vcclab.org/lab/alogps/) (Tetko and Poda, 2004) to obtain bioactive component structures (Supplement 2).

#### 2.2.2 Core Chinese materia medica bioactive components’ targets

Swiss Target Prediction (http://www.swisstargetprediction.ch/) (Gfeller et al., 2014) was used to discover bioactive component targets, with the species confined to “*Homo sapiens*” and a probability value greater than zero. Then, UniProtKB (https://www.uniprot.org/) (UniProt, 2015) standardized the names of targets. (Supplementary 3). The CCMM component-target network was constructed using Cytoscape (http://www.cytoscape.org, version 3.8.0) (Shannon et al., 2003). The degree value of the network was determined using Network Analyzer (Assenov et al., 2008), a Cytoscape plugin.

#### 2.2.3 Targets of male infertility

The term “male infertility” was used to search for MI-related targets in four different databases, including the Online Mendelian Inheritance in Man (OMIM, http://omim.org/) (Amberger and Hamosh, 2017), the Comparative Toxicogenomics Database (CTD, http://ctdbase.org/) (Davis et al., 2019), the DisGeNET database (https://www.disgenet.org/) (Pinero et al., 2017), and GeneCards (https://www.genecards.org/) (Stelzer et al., 2016). The targets were then standardized using the UniProtKB database (Supplement 5).

#### 2.2.4 Core Chinese materia medica-male infertility common-target network

The Venn diagram was used to determine the common targets of CCMM and MI, and then a CCMM-MI common-target network was constructed using the Cytoscape program. Additionally, Network Analyzer was used to determine the network’s topological properties (Supplement 8).

#### 2.2.5 Analyses of gene ontology and kyoto encyclopedia of genes and genomes pathway enrichment

In order to systematically and comprehensively explore the functions and mechanisms of drugs and the pathophysiology of disease, we performed Gene Ontology (GO) enrichment and Kyoto Encyclopedia of Genes and Genomes (KEGG) pathway analyses on the CCMM targets, MI-related targets, and CCMM-MI common targets, respectively. The database is the Database for Annotation Visualization and Integrated Discovery (DAVID, https://david.nicifcrf.gov/, version 6.8) (Sherman and Lempicki, 2009), and the screening criteria is the Bonferroni correction (Chen et al., 2013) (Supplement 4, 6, 9).

#### 2.2.6 PPI network

The PPI parameters for the common targets between CCMM and MI were acquired from the STRING database v11.0 (http://string-db.org) (Szklarczyk et al., 2015), with a confidence level of at least 0.4. We translated the PPI data to TSV format and then created a PPI network using Cytoscape. To filter the core proteins, we used the degree centrality (DC), betweenness centrality (BC), and closeness centrality (CC) topological factors (Supplement 10). Moreover, aim to find the most important functions and signaling pathways, we utilized the BiNGO (http://apps.cytoscape.org/apps/bingo) (Maere et al., 2005) and ClueGO (http://apps.cytoscape.org/apps/cluego) (Bindea et al., 2009) plugins to conduct the enrichment analysis for the core proteins of the CCMM-MI PPI network.

### 2.3 Molecular docking

The Protein Data Bank (PDB) (https://www.rcsb.org/) (Berman et al., 2000) was utilized to get the X-ray crystal structures of the targets, which include AKT1 (PDB ID: 3QKK), MAPK3 (PDB ID: 6GES), MAPK1 (PDB ID: 4QYY), EGFR (PDB ID: 5 × 2K), GAPDH (PDB ID: 6ADE), and TNF (PDB ID: 6ADE) (PDB ID: 1FT4). Next, water molecules and small pro-ligand molecules were removed using PyMOL 2.4 (https://pymol.org/2/) (Yuan et al., 2016). AutoDock Tools 1.5.6 was used to process the protein receptor and ligand files and convert them to pdbqt format. At the center of each grid box was a ligand. Lastly, docking calculations using Autodock Vina 1.1.2 (Trott and Olson, 2010) were conducted. PyMOL 2.4 and ligplus were used to examine and visualize the docking results in 3D and 2D diagrams.

### 2.4 UHPLC-Q-orbitrap HRMS

#### 2.4.1 Preparation of core Chinese materia medica extract

CCMM was supplied by Tongrentang Pharmacy in Beijing (Beijing, China). The following steps were taken to prepare the CCMM extract (Zhang et al., 2012; Lan et al., 2013): (1) After infusing 120 g CCMM samples with 960 ml water for 30 min, they were decocted in a stewpot for 2 h at 100°C. (2) After filtering the solution, the resulting residue was decocted twice for 2 h with 960 ml of water. (3) The filtrate was mixed and concentrated to obtain 1.0 g/ml CCMM extract. (4) For future usage, the CCMM extract was preserved at 4°C.

#### 2.4.2 UHPLC-Q-orbitrap HRMS analysis

The extracts were analyzed using different methods. The instrumentation consisted of an ultimate 3,000 liquid chromatography system coupled to a Q-Orbitrap mass spectrometer equipped with dual ESI/APCI interfaces. An autosampler, a diode array detector, a column compartment, and two pumps comprised the chromatography system. Waters ACQUITY UPLC HSS T3 column (2.1 mm × 100 mm, 1.8 μm; Waters Corporation, Milford, MA, United States) was used for the chromatographic separation. UV detection of UHPLC fractions was performed using a DAD detector with wavelengths ranging from 200 to 400 nm. At a temperature of 30 °C, the analytical column was injected with a volume of 5 L. Gradient elution was performed using 0.1% (v/v) formic acid in water (solvent B) and acetonitrile (solvent A). Kaempferol, quercetin, isorhamnetin, and CCMM extract were analysed in negative mode using an ESI probe. The flow rate was set to 0.3 ml/min, and the gradient elution procedure followed the following: 0–1 min, 95%B; 1–3 min, 95%–85% B; 3–10 min, 85%-75%B; 10–20 min, 75%–68% B, 20–30 min, 68%–50% B; 30–32 min, 50%–0% B; 32–35 min, 0% B; 35–35.1 min, 0%–95% B; 35.1–38 min, 95%B. Beta-sitosterol was analyzed in positive mode using an APCI probe. The flow rate was set to 0.4 ml/min, and the gradient elution procedure followed the following: 0–5 min, 20%–0% B; 5–10 min, 0% B; 10–12 min, 0%–20% B; 12–18 min, 20% B. Positive and negative ion modes were placed in the *m/z* 100–1,500 range for the MS study. Other operating MS characteristics were a sheath gas flow rate of 40 arb, an auxiliary gas flow rate of 15 arb, a capillary temperature of 320°C, an aux gas heater temperature of 350 °C, a positive spray voltage of 3.2 kV, and a negative spray voltage of 3.0 kV. The resolution of the MS is 70,000, while the resolution of the MS/MS is 17,500. Compound Discover and Xcalibur software were used to gather and analyze data. Kaempferol, quercetin, isorhamnetin, and beta-sitosterol standards were purchased from Shanghai yuanye Bio-Technology Co., Ltd.

### 2.5 Experimental validation

#### 2.5.1 Animals

The Vital River Laboratory Animal Technology Co., Ltd. (Beijing, China) provided 64 Kunming male mice weighing 22–25 g. Mice were acclimated to conventional housing circumstances, which included an ambient temperature of 23 ± 2°C, a relative humidity of 23 ± 2°C, and a 12-h light-dark cycle. The Beijing University of Chinese Medicine’s Institutional Animal Care and Use Committee approved the experimental methods and ethics.

#### 2.5.2 A mouse model of MI

The most common causes of MI are spermatogenesis dysfunction and a defect in the testicular (Anawalt, 2013). According to previous experiments (Bakhtiary et al., 2014; Mehraban et al., 2019), we established a MI mice model with sperm quality and testicular pathology that demonstrated the hallmarks of spermatogenesis dysfunction. Intragastrical administration of cyclophosphamide (CP) at a dosage of 60 mg/kg/d for five days was used to simulate MI in mice (Akram et al., 2012; Bakhtiary et al., 2014; Zhao et al., 2015; Yan et al., 2021).

#### 2.5.3 Experimental groups, treatment, and sample preparation

The mice were split into eight groups, each consisting of eight animals. In the normal control (NC) group, physiological saline was consistently given. The remaining seven groups were model control (MC), kaempferol (50 mg/kg), quercetin (50 mg/kg), isorhamnetin (50 mg/kg), beta-sitosterol (50 mg/kg), low-dose, and high-dose CCMM. The present study calculated the low dosage for mice by converting the dose to a human equivalent dose (HED) based on the body surface area of the mice. The mice were given CP intraperitoneally to induce MI. The model control group received physiological saline, the low-dose CCMM group received 10 g/kg CCMM, and the high-dose CCMM group received 20 g/kg CCMM once a day for three weeks. Each group received a single dosage of 20 ml/kg through oral gavage. The mice were weighed and CO_2_ anesthetized following their last treatment, then their left testes and epididymides were taken and weighed using laparotomy.

#### 2.5.4 Sperm quality analysis

Extracts of mice’s epididymal tissue were deposited in a 2 ml Eppendorf tube. After 1 ml M199 (Hyclone; South Logan, UT) was added to the tube, the epididymal tissue was chopped into tiny pieces and placed in a warm water bath set to 37°C for 30 min. Following sperm extraction from the epididymis, the sperms’ quality was assessed using semen analysis equipment and a BK-FL fluorescence microscope (Chongqing Optec Instrument, Chongqing, China). Sperm density (×10^6^/ml), sperm motility (a + b %), sperm viability (%), sperm malformation rate (%), and Lecithin corpuscle density were used as main indicators (Chang et al., 2021).

#### 2.5.5 Histopathological analysis

Tissue from the testes was embedded in paraffin, dried, and stained with hematoxylin and eosin (H&E) after being fixed in 4% paraformaldehyde for 24 h. Cell morphology was observed using a microscope (Jiangsu Kaiji Biotech., China).

#### 2.5.6 Protein extraction and western blot analysis

Testis tissue were lysed in lysis buffer and sonicated. The protein concentration was determined using a BCA protein assay kit (MDL, China). Approximately 30 μg of protein from each sample was separated by 10% sodium dodecyl sulphate-polyacrylamide gel electrophoresis (SDS-PAGE) and transferred to a polyvinylidene fluoride (PVDF) membrane. Membranes were blocked with 5% skimmed milk in TBST and incubated with primary antibodies overnight at 4°C. Antibodies obtained from Abcam (Cambridge, United Kingdom) were as follows: MAPK3/1 (ERK1/2) (ab184699), TNF-α (ab205587). Antibodies purchased from Cell Signaling Technology (Danvers, MA, United States) included those against Akt (2938S) and EGFR (2646S). GAPDH (MDL, China) was regarded as the internal reference. Membranes were incubated with the corresponding secondary antibody (MDL, China) for 1 h at room temperature and washed in TBST. Protein signals were detected using ECL Detection Reagent kit (MDL, China). Images were captured by ChemiDoc MP Imaging System (Bio-rad, United States).

#### 2.5.7 Statistical analysis

All of the data is presented as mean ± SD. One-way analysis of variance (ANOVA) with GraphPad software (version 8.0) was used to perform comparisons of multiple groups and pairwise comparisons. Statistical significance was defined as *p* < 0.05.

## 3 Results

### 3.1 Data mining

**FIGURE 1:**
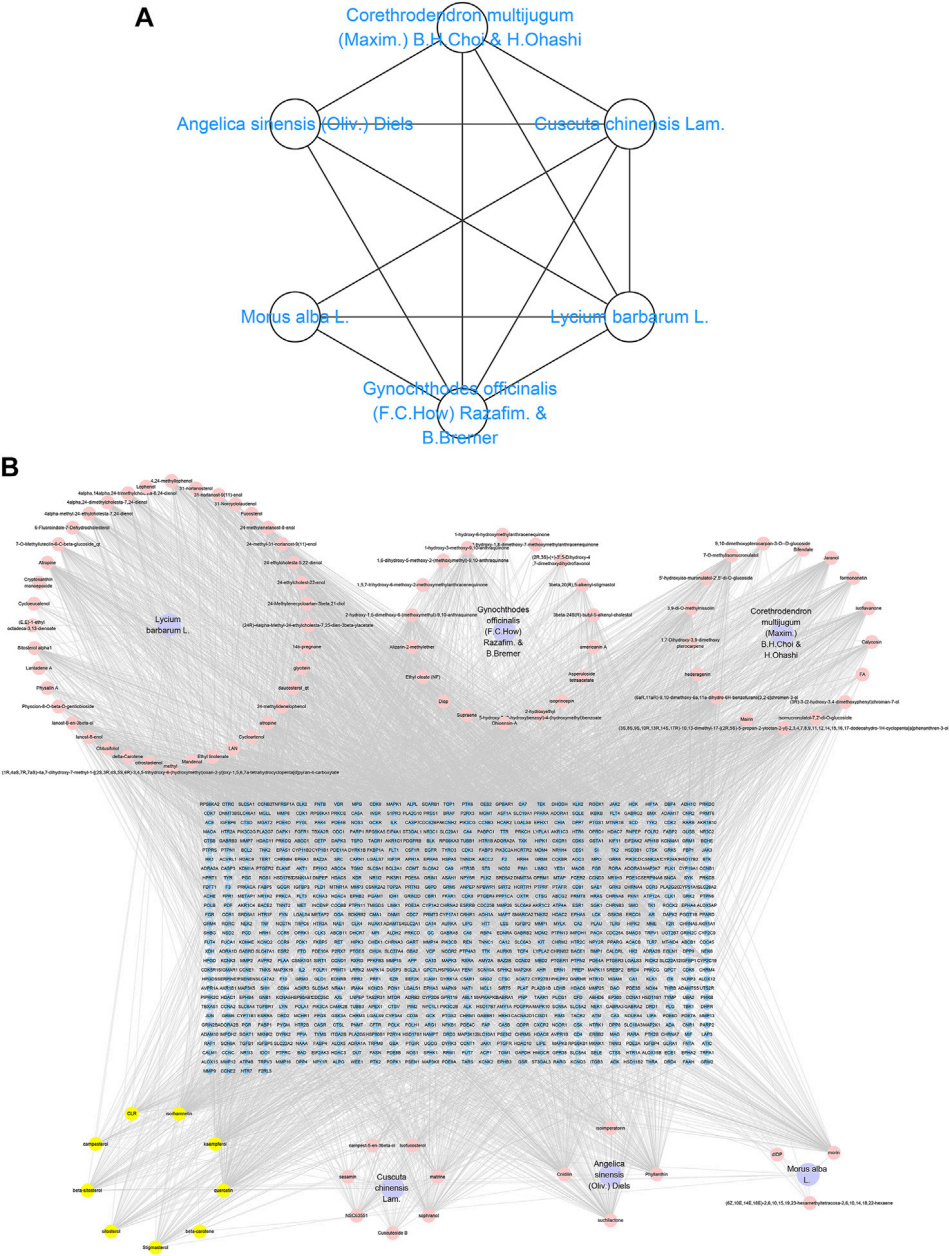
CCMM and component-target network. **(A)** CCMM network. **(B)** CCMM component-target network. Purple nodes stand for herbs of CCMM. Pink nodes represent bioactive components from each herb. Yellow nodes indicate bioactive components that appear more than once from different herbs. Blue nodes stand for targets.

We collected 289 TCM prescriptions and 149 CMM used to treat MI. The top 20 high frequent CMM of the prescriptions are shown in Table 1, including Gynochthodes officinalis (F.C.How) Razafim. and B. Bremer, Cuscuta chinensis Lam., Lycium barbarum L., Morus alba L., Angelica sinensis (Oliv.) Diels, Corethrodendron multijugum (Maxim.) B.H.Choi and H. Ohashi, Swim bladder, Plantago ovata Forssk., *Terminalia* chebula Retz., *Cyperus* rotundus L., Grona styracifolia (Osbeck) H. Ohashi and K. Ohashi, Hordeum vulgare L., Endothelium corneum, Crataegus monogyna Jacq., *Glycine* max (L.) Merr., *Hippocampus* japonicus, Fraxinus excelsior L., *Tragopogon* porrifolius L., Placenta Hominis, and Panax ginseng C.A.Mey. The frequency of the top six CMM is greater than 170, with a percentage greater than 60%, indicating that they are CCMM in the prescriptions on MI. The combinations of CCMM are shown in Table 2. In addition, the application mode of CCMM was virtualized as a network using the TCMISS software (Figure 1A).

**TABLE 1:** The top 20 high frequent CMM in TCM prescriptions.

Number	Name of CMM	Frequency	Percentage (%)
1	Gynochthodes officinalis (F. C. How) Razafim. and B.Bremer	225	77.85
2	Cuscuta chinensis Lam	208	71.97
3	Lycium barbarum L	194	67.13
4	Morus alba L	188	65.05
5	Angelica sinensis (Oliv.) Diels	183	63.32
6	Corethrodendron multijugum (Maxim.) B. H. Choi and H.Ohashi	176	60.90
7	Swim bladder	156	53.98
8	Plantago ovata Forssk	123	42.56
9	*Terminalia* chebula Retz	112	38.75
10	*Cyperus* rotundus L	108	37.37
11	Grona styracifolia (Osbeck) H.Ohashi and K.Ohashi	71	24.57
12	Hordeum vulgare L	68	23.53
13	Endothelium corneum	54	18.69
14	Crataegus monogyna Jacq	43	14.88
15	*Glycine* max (L.) Merr	40	13.84
16	*Hippocampus* japonicus	34	11.76
17	Fraxinus excelsior L	33	11.42
18	*Tragopogon* porrifolius L	31	10.73
19	Placenta Hominis	29	10.03
20	Panax ginseng C. A.Mey	24	8.30

**TABLE 2:** The combinations of CCMM in TCM prescriptions.

Number	The combinations of CCMM	Frequency
1	Cuscuta chinensis Lam., Gynochthodes officinalis (F. C. How) Razafim. and B. Bremer	188
2	Cuscuta chinensis Lam., Lycium barbarum L	178
3	Cuscuta chinensis Lam., Angelica sinensis (Oliv.) Diels	153
4	Cuscuta chinensis Lam., Morus alba L	165
5	Cuscuta chinensis Lam., Corethrodendron multijugum (Maxim.) B. H. Choi and H. Ohashi	153
6	Gynochthodes officinalis (F. C. How) Razafim. and B. Bremer, Lycium barbarum L	176
7	Gynochthodes officinalis (F. C. How) Razafim. and B. Bremer, Angelica sinensis (Oliv.) Diels	170
8	Gynochthodes officinalis (F. C. How) Razafim. and B.Bremer, Morus alba L	167
9	Corethrodendron multijugum (Maxim.) B.H.Choi and H.Ohashi, Gynochthodes officinalis (F. C. How) Razafim. and B. Bremer	167
10	Angelica sinensis (Oliv.) Diels, Lycium barbarum L	148
11	Lycium barbarum L., Morus alba L	155
12	Corethrodendron multijugum (Maxim.) B. H. Choi and H. Ohashi, Lycium barbarum L	148
13	Corethrodendron multijugum (Maxim.) B. H. Choi and H. Ohashi, Angelica sinensis (Oliv.) Diels	171
14	Cuscuta chinensis Lam., Gynochthodes officinalis (F. C. How) Razafim. and B.Bremer, Lycium barbarum L	163
15	Cuscuta chinensis Lam., Gynochthodes officinalis (F. C. How) Razafim. and B. Bremer, Angelica sinensis (Oliv.) Diels	148
16	Cuscuta chinensis Lam., Gynochthodes officinalis (F. C. How) Razafim. and B. Bremer, Morus alba L	148
17	Cuscuta chinensis Lam., Corethrodendron multijugum (Maxim.) B.H.Choi and H.Ohashi, Gynochthodes officinalis (F. C. How) Razafim. and B. Bremer	148
18	Cuscuta chinensis Lam., Lycium barbarum L., Morus alba L	143
19	Cuscuta chinensis Lam., Corethrodendron multijugum (Maxim.) B. H. Choi and H. Ohashi, Angelica sinensis (Oliv.) Diels	149
20	Gynochthodes officinalis (F. C. How) Razafim. and B. Bremer, Angelica sinensis (Oliv.) Diels, Lycium barbarum L	143
21	Gynochthodes officinalis (F. C. How) Razafim. and B. Bremer, Lycium barbarum L., Morus alba L	141
22	Corethrodendron multijugum (Maxim.) B. H. Choi and H. Ohashi, Gynochthodes officinalis (F. C. How) Razafim. and B. Bremer, Lycium barbarum L	143
23	Corethrodendron multijugum (Maxim.) B. H. Choi and H.Ohashi, Gynochthodes officinalis (F. C. How) Razafim. and B. Bremer, Angelica sinensis (Oliv.) Diels	163
24	Corethrodendron multijugum (Maxim.) B. H. Choi and H. Ohashi, Angelica sinensis (Oliv.) Diels, Lycium barbarum L	146
25	Cuscuta chinensis Lam., Corethrodendron multijugum (Maxim.) B. H. Choi and H. Ohashi, Gynochthodes officinalis (F. C. How) Razafim. and B. Bremer, Angelica sinensis (Oliv.) Diels	144
26	Corethrodendron multijugum (Maxim.) B. H. Choi and H. Ohashi, Gynochthodes officinalis (F. C. How) Razafim. and B. Bremer, Angelica sinensis (Oliv.) Diels, Lycium barbarum L	141

### 3.2 Network pharmacology

#### 3.2.1 Core Chinese materia medica component-target network

TCMSP, TCMID, and Swiss Target Prediction databases were used to gather information on the components and targets of CCMM in the treatment of MI. A total of 20 components and 359 targets form Gynochthodes officinalis (F.C.How) Razafim. and B. Bremer, 13 components and 302 targets from Cuscuta chinensis Lam., 47 components and 446 targets from Lycium barbarum L., seven components and 157 targets from Morus alba L., six components and 296 targets from Angelica sinensis (Oliv.) Diels, 22 components and 436 targets from Hedysarum Multijugum Maxim, were obtained. Then, we constructed a CCMM component-target network using 98 components and 816 targets (Figure 1B). We found that beta-sitosterol, sitosterol, quercetin, kaempferol, isorhamnetin, CLR, campesterol, Stigmasterol, and beta-carotene are repeated more than once in CCMM. The structure, OB, and DL of these duplicate components are shown in Table 3. So, we thought that these duplicate components should be further explored in the following experiment.

**TABLE 3:** The components with more than one occurrence in different herbs of CCMM in TCM prescriptions.

Mol ID	Molecule name	Structure	OB (%)	DL	Herb
MOL000358	Beta-sitosterol	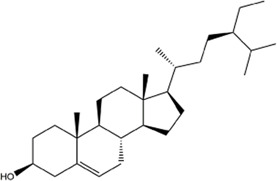	36.91	0.75	Gynochthodes officinalis (F.C.How) Razafim. and B.Bremer
					Cuscuta chinensis Lam
					Lycium barbarum L
					Morus alba L
					Angelica sinensis (Oliv.) Diels
					Corethrodendron multijugum (Maxim.) B.H.Choi and H.Ohashi
MOL000359	sitosterol	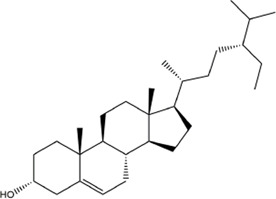	36.91	0.75	Gynochthodes officinalis (F.C.How) Razafim. and B.Bremer
					Lycium barbarum L
					Corethrodendron multijugum (Maxim.) B.H.Choi and H.Ohashi
MOL000098	quercetin	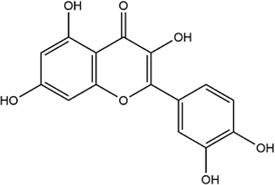	46.43	0.28	Cuscuta chinensis Lam
					Lycium barbarum L
					Morus alba L
					Corethrodendron multijugum (Maxim.) B.H.Choi and H.Ohashi
MOL000422	kaempferol	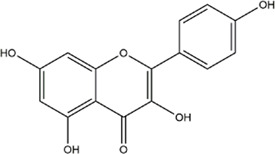	41.88	0.24	Cuscuta chinensis Lam
					Morus alba L
					Corethrodendron multijugum (Maxim.) B.H.Choi and H.Ohashi
MOL000354	isorhamnetin	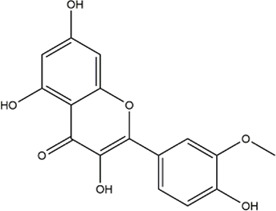	49.6	0.31	Cuscuta chinensis Lam
					Corethrodendron multijugum (Maxim.) B.H.Choi and H.Ohashi
MOL000953	CLR	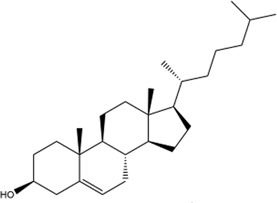	37.87	0.68	Cuscuta chinensis Lam
					Lycium barbarum L
MOL005438	campesterol	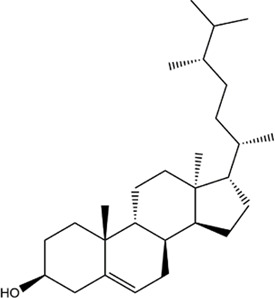	37.58	0.71	Cuscuta chinensis Lam
					Lycium barbarum L
MOL000449	Stigmasterol	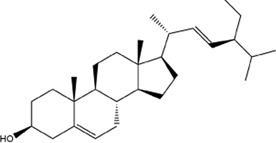	43.83	0.76	Lycium barbarum L
					Angelica sinensis (Oliv.) Diels
MOL002773	beta-carotene	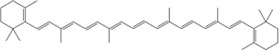	37.18	0.58	Lycium barbarum L
					Morus alba L

#### 3.2.2 Gene Ontology and kyoto Encyclopedia of Genes and Genomes enrichment analyses of the CCMM targets

**FIGURE 2:**
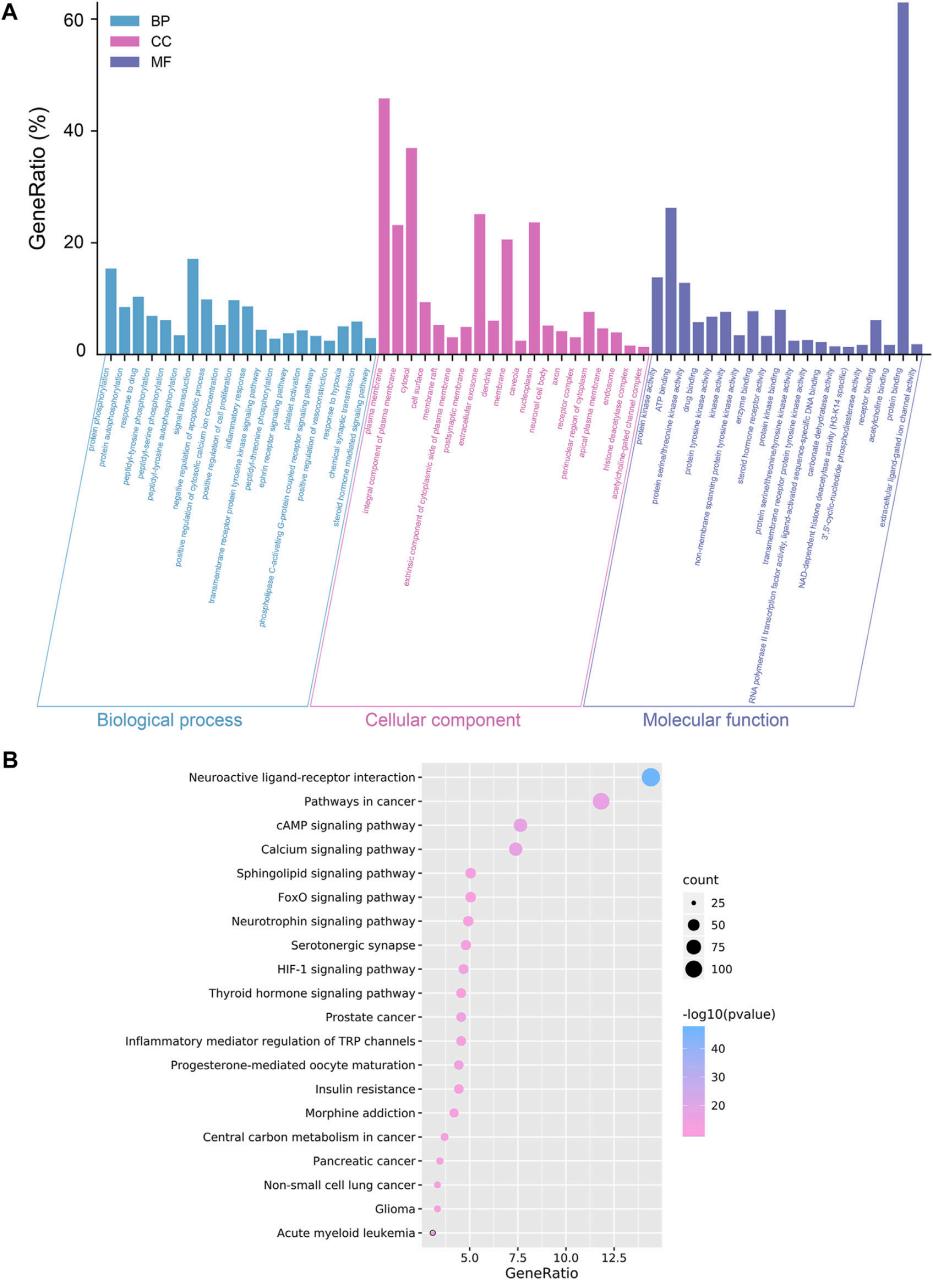
Analyses of GO and KEGG enrichment for CCMM targets (*p*-value ≤ 0.05). **(A)** The top 20 GO enrichment terms include biological process, cellular component, and molecular function. The GeneRatio (%) of the top 20 most significant enrichments is shown in the bar graphs. **(B)** The top 20 KEGG pathways. The color grades reflect the various *p*-value thresholds, and the sizes of the dots indicate the number of targets associated with each term.

The investigation of GO enrichment is divided into three parts: biological process, cellular component, and molecular function. We found that CCMM could inhibit apoptosis, promote cell proliferation, and regulate the cytosolic calcium ion concentration through negative regulation of apoptotic process (GO:0043066), positive regulation of cytosolic calcium ion concentration (GO:0007204), positive regulation of cell proliferation (GO:0008284). Additionally, Prostate cancer (hsa05215), HIF-1 signaling pathway (hsa04066), Progesterone-mediated oocyte maturation (hsa04914), and Acute myeloid leukemia (hsa05221) are related to male reproductive function, which was shown in Figure 2.

#### 3.2.3 MI-related targets

**FIGURE 3:**
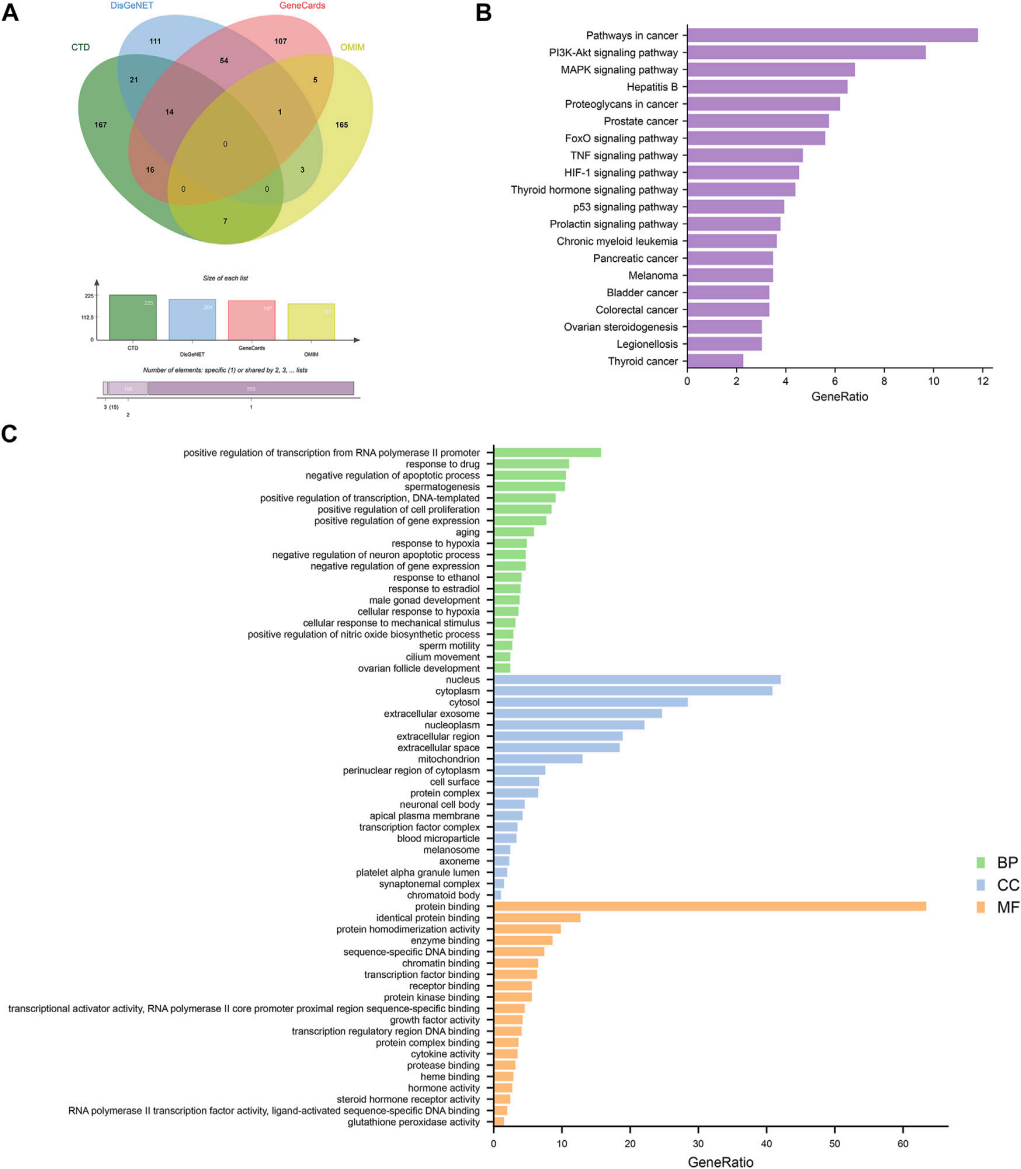
Analyses of GO and KEGG enrichment for MI-related targets (*p*-value ≤ 0.05). **(A)** Venn diagram: the number of MI-related targets from the four different databases are 225, 204, 197, and 181. **(B)** The top 20 KEGG pathways. **(C)** The top 20 GO enrichment terms include biological process, cellular component, and molecular function. The GeneRatio (%) of the top 20 most significant enrichments is shown in the bar graphs.

A total of 671 targets of MI were collected from four different databases. Among these, 225 targets were from CTD, 210 targets were from DisGeNET, 197 targets were from GeneCards, 181 targets were from OMIM (Figure 3A). Following that, we conducted GO and KEGG enrichment analysis on MI-related targets (Figures 3B,C). The results showed that pathways in cancer (hsa05200), PI3K-Akt signaling pathway (hsa04151), MAPK signaling pathway (hsa04010) were the most significant signaling pathways. Moreover, positive regulation of transcription from RNA polymerase II promoter (GO:0045944), response to drug (GO:0042493), negative regulation of apoptotic process (GO:0043066), spermatogenesis (GO:0007283) were the most significant terms in biological process (BP). According to the above results, we suggest that MI is related to apoptosis and spermatogenesis.

#### 3.2.4 Core Chinese materia medica-MI common-target network

**FIGURE 4:**
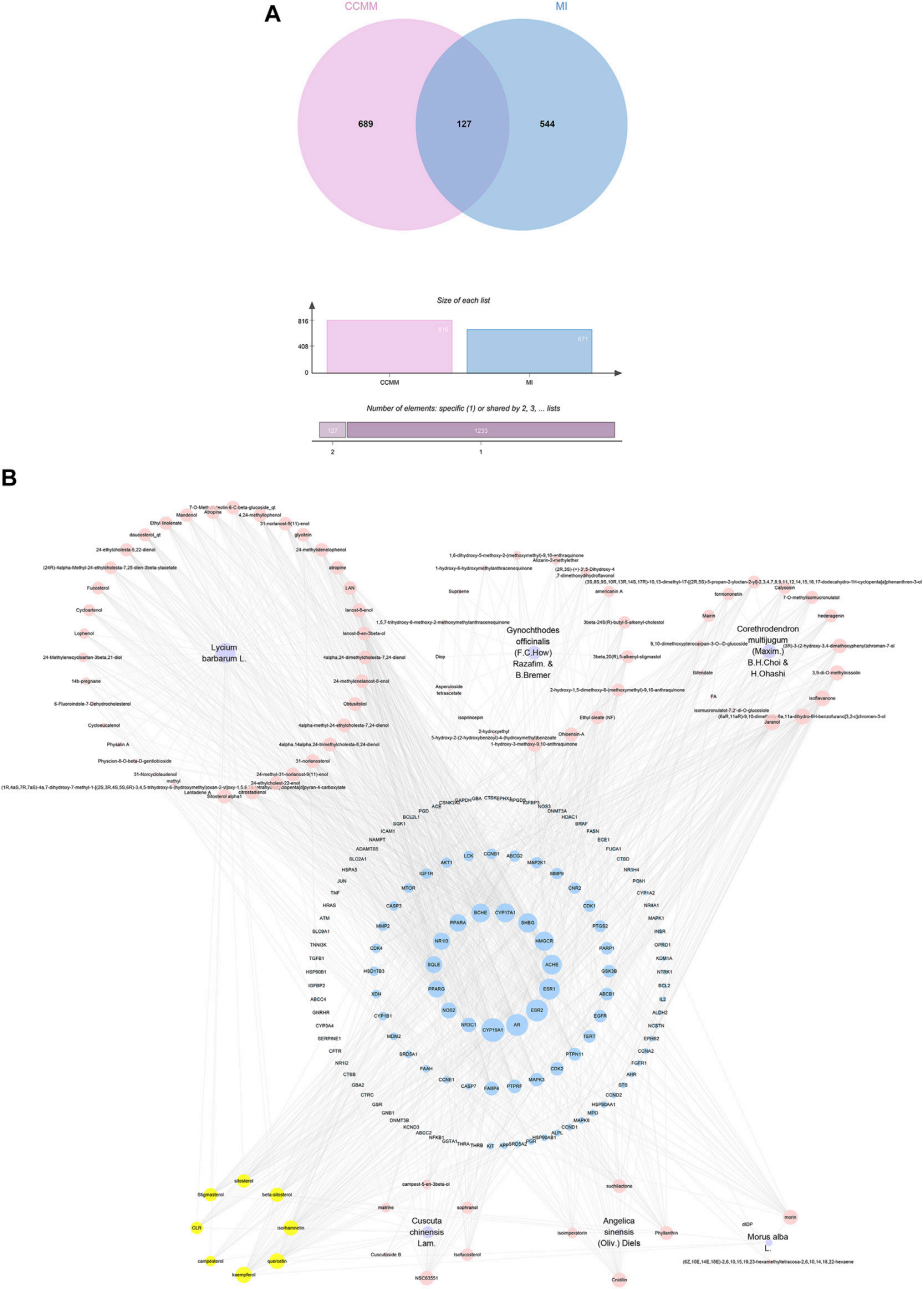
CCMM-MI common targets and network. **(A)** The Venn diagram’s intersection: CCMM and MI overlap 127 targets. **(B)** CCMM-MI common-target network. Purple nodes stand for herbs of CCMM. Pink nodes represent bioactive components from each herb. Yellow nodes indicate bioactive components that appear more than once from different herbs. Blue nodes stand for the common targets of CCMM and MI. The diameter of the circle denotes the target protein’s node degree.

Based on the results of the Venn diagram, we obtained 127 common targets of CCMM and MI (Figure 4A). Then, we established a CCMM-MI common-target network, including 90 components and 127 targets (Figure 4B). Especially, it suggested that Jaranol, kaempferol, (6aR,11aR)-9,10-dimethoxy-6a, 11a-dihydro-6H-benzofurano [3,2-c]chromen-3-ol, isoflavanone, quercetin, 1-hydroxy-3-methoxy-9,10-anthraquinone, 3,9-di-O-methylnissolin, isorhamnetin, morin, (3R)-3-(2-hydroxy-3,4-dimethoxyphenyl) chroman-7-ol, Cnidilin, Sitosterol alpha1, citrostadienol, beta-sitosterol, and NSC63551 are the top 15 components with high degree value in the process of CCMM against MI. The structure and degree value of these components are shown in Table 4. To be noted, kaempferol, quercetin, isorhamnetin, and beta-sitosterol were the duplicate components in CCMM. As shown in Table 5, Aromatase (CYP19A1), Androgen receptor (AR), Estrogen receptor beta (ESR2), Estrogen receptor (ESR1), Acetylcholinesterase (ACHE), 3-hydroxy-3-methylglutaryl-coenzyme A reductase (HMGCR), Sex hormone-binding globulin (SHBG), Steroid 17-alpha-hydroxylase/17,20 lyase (CYP17A1), Cholinesterase (BCHE), Peroxisome proliferator-activated receptor alpha (PPARA), Nuclear receptor subfamily 1 group I member 3 (NR1I3), Squalene monooxygenase (SQLE), Peroxisome proliferator-activated receptor gamma (PPARG), Nitric oxide synthase, inducible (NOS2), and Glucocorticoid receptor (NR3C1) were the top 15 CCMM-MI common targets with high degree value.

**TABLE 4:** The top 15 components with high degree value acted on the CCMM-MI common targets.

Number	Molecule name	Structure	Degree	Herb
1	Jaranol	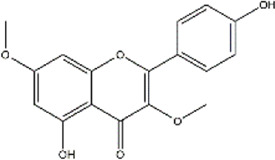	28	Corethrodendron multijugum (Maxim.) B.H.Choi and H.Ohashi
2	kaempferol	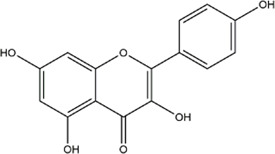	28	Cuscuta chinensis Lam
				Morus alba L
				Corethrodendron multijugum (Maxim.) B.H.Choi and H.Ohashi
3	(6aR,11aR)-9,10-dimethoxy-6a,11a-dihydro-6H-benzofurano [3,2-c]chromen-3-ol	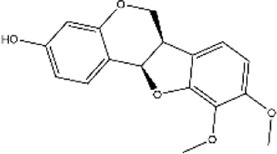	27	Corethrodendron multijugum (Maxim.) B.H.Choi and H.Ohashi
4	isoflavanone	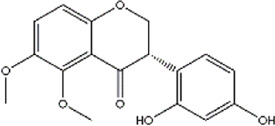	27	Corethrodendron multijugum (Maxim.) B.H.Choi and H.Ohashi
5	quercetin	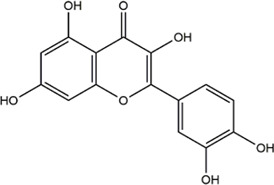	26	Cuscuta chinensis Lam
				Lycium barbarum L
				Morus alba L
				Corethrodendron multijugum (Maxim.) B.H.Choi and H.Ohashi
6	1-hydroxy-3-methoxy-9,10-anthraquinone	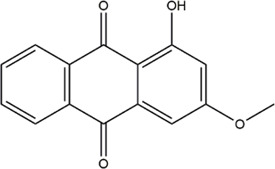	25	Gynochthodes officinalis (F.C.How) Razafim. and B.Bremer
7	3,9-di-O-methylnissolin	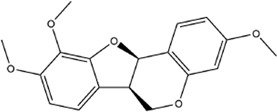	24	Corethrodendron multijugum (Maxim.) B.H.Choi and H.Ohashi
8	isorhamnetin	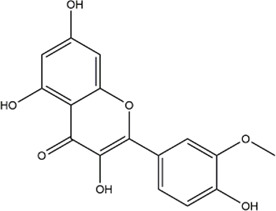	24	Cuscuta chinensis Lam
				Corethrodendron multijugum (Maxim.) B.H.Choi and H.Ohashi
9	morin	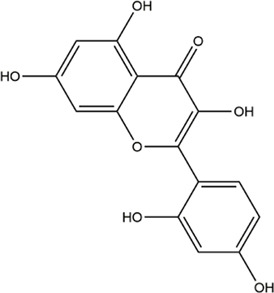	23	Morus alba L
10	(3R)-3-(2-hydroxy-3,4-dimethoxyphenyl)chroman-7-ol	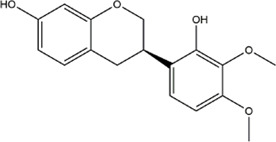	22	Corethrodendron multijugum (Maxim.) B.H.Choi and H.Ohashi
11	Cnidilin	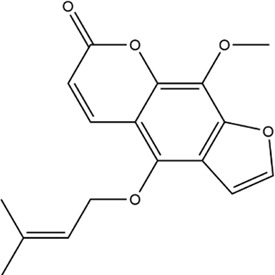	22	Angelica sinensis (Oliv.) Diels
12	Sitosterol alpha1	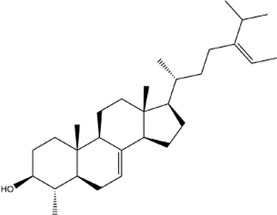	21	Lycium barbarum L
13	citrostadienol	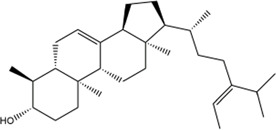	21	Lycium barbarum L
14	beta-sitosterol	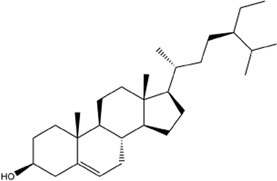	21	Gynochthodes officinalis (F.C.How) Razafim. and B.Bremer
				Cuscuta chinensis Lam
				Lycium barbarum L
				Morus alba L
				Angelica sinensis (Oliv.) Diels
				Corethrodendron multijugum (Maxim.) B.H.Choi and H.Ohashi
15	NSC63551	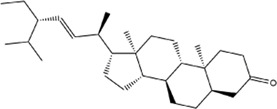	21	Cuscuta chinensis Lam

**TABLE 5:** The top 15 CCMM-MI common targets with high degree value.

Number	Gene name	Protein name	Degree
1	CYP19A1	Aromatase	56
2	AR	Androgen receptor	50
3	ESR2	Estrogen receptor beta	50
4	ESR1	Estrogen receptor	46
5	ACHE	Acetylcholinesterase	42
6	HMGCR	3-hydroxy-3-methylglutaryl-coenzyme A reductase	40
7	SHBG	Sex hormone-binding globulin	40
8	CYP17A1	Steroid 17-alpha-hydroxylase/17,20 lyase	39
9	BCHE	Cholinesterase	38
10	PPARA	Peroxisome proliferator-activated receptor alpha	33
11	NR1I3	Nuclear receptor subfamily 1 group I member 3	32
12	SQLE	Squalene monooxygenase	31
13	PPARG	Peroxisome proliferator-activated receptor gamma	31
14	NOS2	Nitric oxide synthase, inducible	27
15	NR3C1	Glucocorticoid receptor	22

#### 3.2.5 Gene Ontology and kyoto Encyclopedia of Genes and Genomes pathway enrichment analyses of the core Chinese materia medica-MI common targets

**FIGURE 5:**
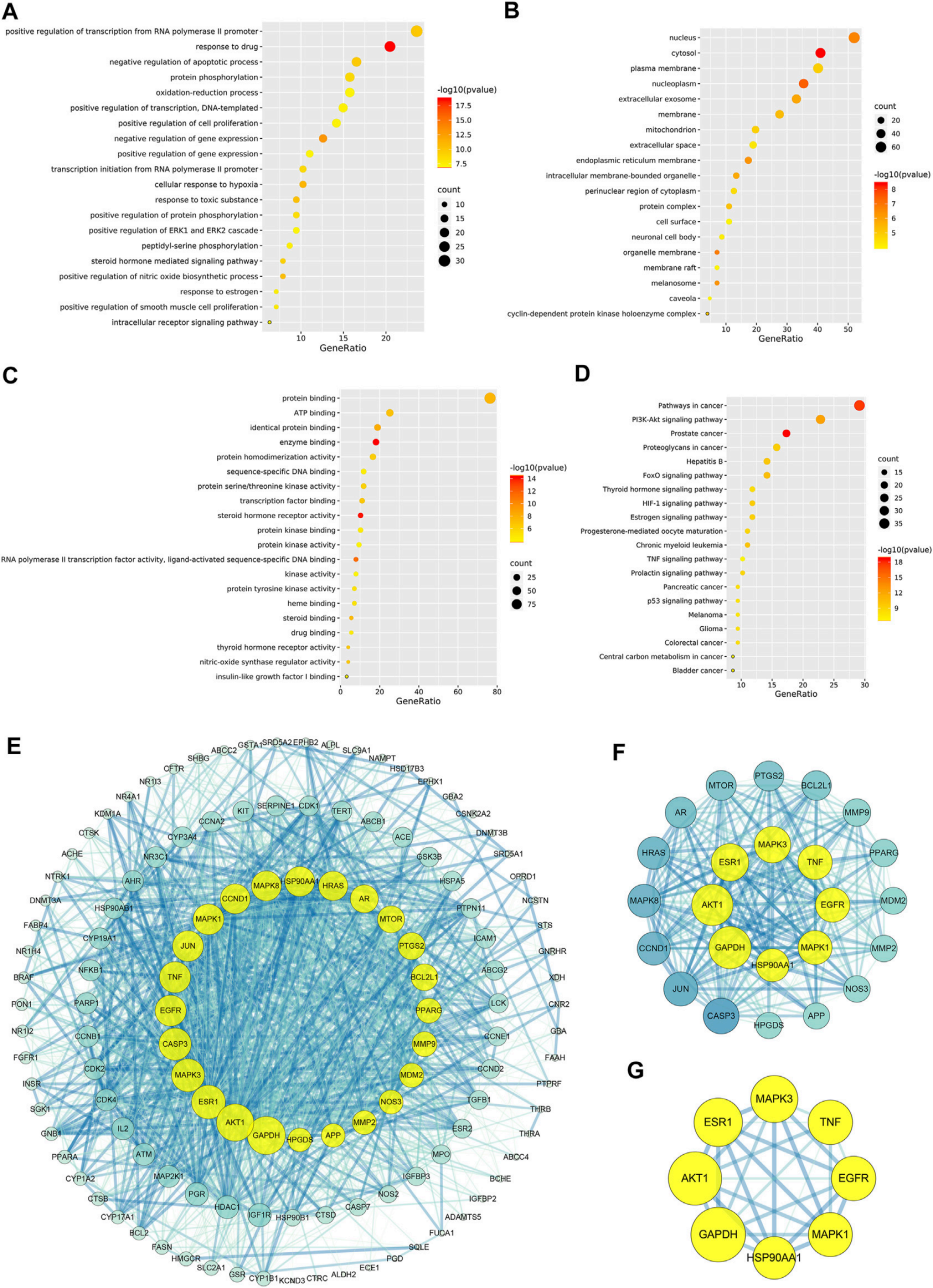
The enrichment and PPI analyses of the CCMM-MI common targets (*p*-value ≤ 0.05). **(A)** The twenty most important biological processes. **(B)** A total of nineteen cell components. **(C)** The twenty most important molecular functions. **(D)** A list of the top twenty KEGG pathways. The color grades represent various *p*-value thresholds, and the sizes of the dots indicate the number of targets correlated with each phrase. **(E)** The PPI network of CCMM and MI. **(F)** The PPI network by the screening criteria of DC ≥ 41. **(G)** DC ≥ 60, BC ≥ 0.025, and CC ≥ 0.651 were used as screening criteria for the PPI network. The node’s size and color indicate the degree of the target protein. The width and color of the edge indicate the target protein’s combined score.

Figure 5A indicated that CCMM could inhibit apoptosis in the treatment of MI via negative regulation of apoptotic process (GO:0043066). In addition, CCMM can reduce oxidant stress through oxidation-reduction process (GO:0055114), and positive regulation of nitric oxide biosynthetic process (GO:0045429). CCMM can also promote cell proliferation for treating MI, through positive regulation of cell proliferation (GO:0008284). Besides, CCMM can regulate male reproductive function on MI, through positive regulation of ERK1 and ERK2 cascade (GO:0070374). As shown in Figures 5B,C, CCMM mainly regulated hormones in the treatment of MI, *via* steroid hormone receptor activity (GO:0003707), and thyroid hormone receptor activity (GO:0004887). The main KEGG signaling pathways were PI3K-Akt signaling pathway, Estrogen signaling pathway, HIF-1 signaling pathway, and TNF signaling pathway, demonstrating that CCMM could treat MI *via* regulating hormones, reducing apoptosis, oxidant stress, and inflammatory (Figure 5D).

#### 3.2.6 PPI network analysis

A PPI network was created to evaluate the CCMM-MI common targets’ core proteins (Figures 5E–G). Under the screening criteria of DC ≥ 60, BC ≥ 0.025, and CC ≥ 0.651, Glyceraldehyde-3-phosphate dehydrogenase (GAPDH), RAC-alpha serine/threonine-protein kinase (AKT1), Estrogen receptor (ESR1), Mitogen-activated protein kinase 3 (MAPK3), Epidermal growth factor receptor (EGFR), Tumor necrosis factor (TNF), Mitogen-activated protein kinase 1 (MAPK1), Heat shock protein HSP 90-alpha (HSP90AA1) are the core proteins in the PPI network (Figure 5G).

#### 3.2.7 Gene Ontology and kyoto Encyclopedia of Genes and Genomes pathway enrichment analyses of the core proteins

**FIGURE 6:**
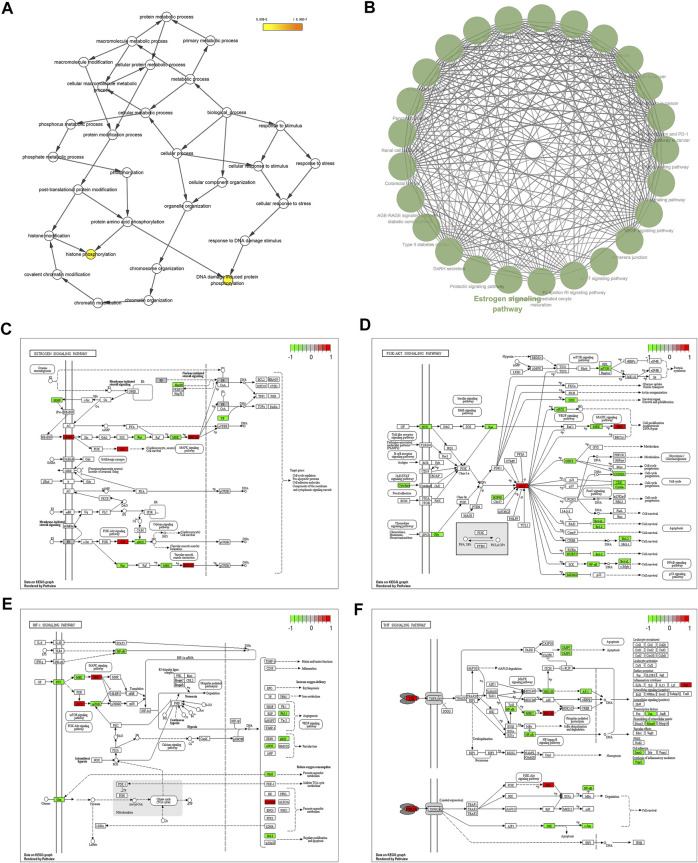
Analyses of GO and KEGG enrichment for core proteins (*p*-value ≤ 0.05). **(A)** GO enrichment for core proteins using BiNGO. Biological process, cellular component, and molecular function are all included. **(B)** KEGG pathway enrichment of the core proteins using ClueGO. **(C)** Estrogen signaling pathway. **(D)** PI3K-Akt signaling pathway. **(E)** HIF-1 signaling pathway. **(F)** TNF signaling pathway. The green rectangle represents the CCMM-MI common-target associated with the main KEGG signaling pathways. The red rectangle represents the key targets of CCMM on MI.

Furthermore, GO and KEGG enrichment analyses were conducted on the core proteins of the PPI network. The results showed that DNA damage induced protein phosphorylation and histone phosphorylation are the most significant GO terms (Figure 6A). The result of GlueGO indicated that Estrogen signaling pathway has the most significant pvalue (6.0 × 10^−10^) and the largest number of associated core proteins (AKT1, EGFR, ESR1, HSP90AA1, MAPK1, and MAPK3). Therefore, Estrogen signaling pathway is the most significant KEGG signaling pathway (Figure 6B). As shown in Figures 6C–F, the green rectangle represents the CCMM-MI common-target associated with the main KEGG signaling pathways (PI3K-Akt signaling pathway, Estrogen signaling pathway, HIF-1 signaling pathway, TNF signaling pathway). The red rectangle represents six overlapping targets (AKT1, MAPK3, MAPK1, EGFR, GAPDH, and TNF) between four main KEGG signaling pathways and eight core proteins (GAPDH, AKT1, ESR1, MAPK3, EGFR, TNF, MAPK1, and HSP90AA1). The overlapping targets are the key targets of CCMM on MI (Table 6).

**TABLE 6:** The most significant KEGG signaling pathways and key targets.

Classification	KEGG signaling pathway	Key targets
Hormone regulation	Estrogen signaling pathway	AKT1 (Akt)
		MAPK3/1 (ERK1/2)
		EGFR
Apoptosis	PI3K-Akt signaling pathway	AKT1 (AKT)
		MAPK3/1 (ERK)
Oxidant stress	HIF-1 signaling pathway	AKT1 (AKT)
		MAPK3/1 (ERK)
		GAPDH
Inflammatory	TNF signaling pathway	AKT1 (Akt)
		MAPK3/1 (ERK1/2)
		TNF

### 3.3 Molecular docking

**FIGURE 7:**
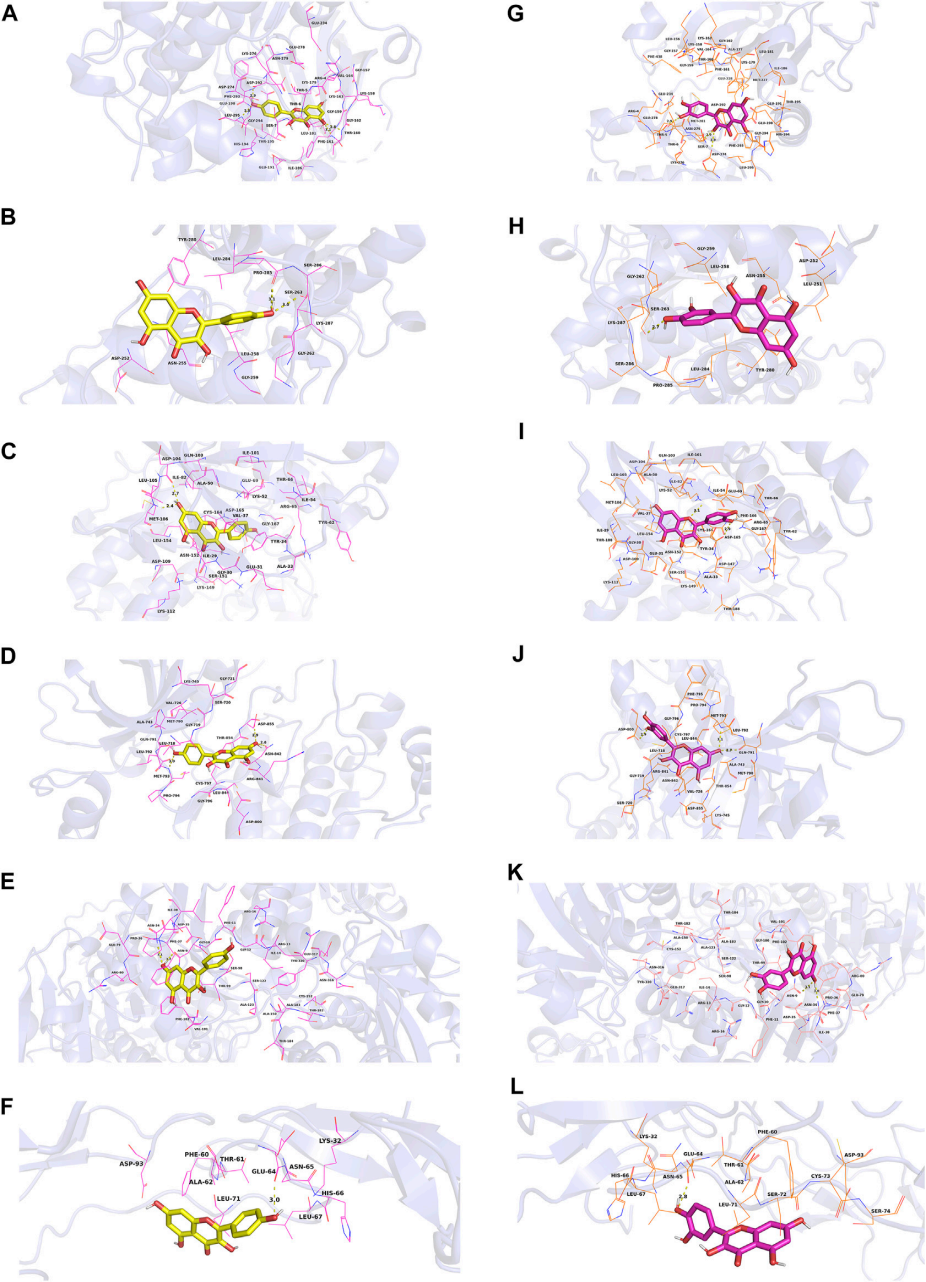
Molecular models of kaempferol and quercetin that bind to the predicted protein targets, shown as 3D diagrams. **(A)** Kaempferol-AKT1, **(B)** kaempferol-MAPK3, **(C)** kaempferol-MAPK1, **(D)** kaempferol-EGFR, **(E)** kaempferol-GAPDH, **(F)** kaempferol-TNF, **(G)** quercetin-AKT1, **(H)** quercetin-MAPK3, **(I)** quercetin-MAPK1, **(J)** quercetin-EGFR, **(K)** quercetin-GAPDH, (L) quercetin-TNF.

In this study, we chose kaempferol, quercetin, isorhamnetin, and beta-sitosterol from CCMM as small molecules (ligands), AKT1, MAPK3, MAPK1, EGFR, GAPDH, and TNF as proteins to perform the molecular docking (Table 7). In AKT1, kaempferol was conjugated with sixteen residues to generate hydrophobic interactions (Gly 294, Leu181, Gly162, Lys179, Gly159, Asp274, and Ser7) and 4 hydrogen bonds [kaempferol_O5_: Asp292_O_ (2.9 Å), kaempferol_O5_: Leu195_N_ (2.8 Å), kaempferol_O4_: Phe161_N_ (3.1 Å), kaempferol_O4_: Thr160_N_ (2.8 Å)] (Figure 7A,9A). Additionally, it was predicted that kaempferol could interact with MAPK3 through Gly262, Leu284, Asn255, Leu258, Gly259, Lys287 and form two hydrogen bonds with the residues Pro285 (3.1 Å) and Ser263 (3.0 Å) (Figure 7B,9B). Kaempferol interacted with MAPK1 through hydrophobic interactions with adjacent residues Asp165, Ala50, Leu105, Leu154, Ile29, Val137, Lys52, Tyr34, and two hydrogen bonds are formed Asp104 (2.7 Å) and Met106 (3.1 Å) (Figure 7C,9C). Additionally, kaempferol is linked to a pocket in EGFR, containing Ala743, Leu792, Leu718, Cys797, Arg841, Leu844, and Gly796. Kaempferol_O5_ and Met793_N_ (3.0 Å), kaempferol_O6_ and Asp855_OD2_ (2.9 Å), kaempferol_O6_ and Asn842_OD1_ (2.8 Å) formed the hydrogen bonds, so the interaction between the ligand and the EGFR protein was strengthened. (Figures 7D,9D). Moreover, kaempferol was bound to GAPDH via adjacent residues (Ile38, Phe37, Thr99, Val101, Phe102, Arg80, Pro36, Asp35, and Gly12) and two hydrogens bonds with Asn34 (3.1 Å) and Asn9 (3.2 Å) (Figures 7E,9E). Kaempferol was also predicted to interact with TNF via Leu67, Ala62, Phe60, Leu71, Asn65 and form a hydrogen bond with the residue Glu64 (3.0 Å) (Figures 7F,9F).

**TABLE 7:** The binding energy of molecular docking between ligands and proteins.

Ligand	Proteins	Affinity	Dist from best mode	
		(kcal/mol)	rmsd l.b	rmsd u.b
kaempferol	AKT1	−7.5	0.000	0.000
	MAPK3	−5.4	0.000	0.000
	MAPK1	−8.1	0.000	0.000
	EGFR	−6.6	0.000	0.000
	GAPDH	−6.5	0.000	0.000
	TNF	−4.4	0.000	0.000
quercetin	AKT1	−7.7	0.000	0.000
	MAPK3	−5.3	0.000	0.000
	MAPK1	−8.1	0.000	0.000
	EGFR	−6.5	0.000	0.000
	GAPDH	−6.5	0.000	0.000
	TNF	−4.3	0.000	0.000
isorhamnetin	AKT1	−7.5	0.000	0.000
	MAPK3	−5.4	0.000	0.000
	MAPK1	−8.1	0.000	0.000
	EGFR	−6.7	0.000	0.000
	GAPDH	−6.6	0.000	0.000
	TNF	−4.4	0.000	0.000
beta-sitosterol	AKT1	−9.8	0.000	0.000
	MAPK3	−5.3	0.000	0.000
	MAPK1	−9.5	0.000	0.000
	EGFR	−7.2	0.000	0.000
	GAPDH	−7.5	0.000	0.000
	TNF	−5.9	0.000	0.000

As shown in Figure 7G and Figure 9G quercetin was observed to interact with AKT1 via Lys276, Glu278, Leu295, Gly294, His194, Phe161, Glu191, Ile186, and Asp292 and form 3 hydrogen bonds with Thr5 (2.9 Å), Asp274 (2.9 Å), and Ser7 (2.9 Å). According to the analysis results shown in Figure 7H and Figure 9H, quercetin forms hydrophobic bonds with seven residues in MAPK3 (Leu258, Gly262, Leu284, Asn255, Gly259, Lys287, and Pro285) and a hydrogen bond (quercetin_O4_: Ser263_OG_ (2.7 Å)). Figure 7I and Figure 9I demonstrate that quercetin was predicted to interact with MAPK1 via Gly167, Tyr34, Gln103, Ala50, Val37, Ile54, Glu69, and Thr66, and formed two hydrogen bonds with Asp165 (2.9 Å) and Lys52 (3.1 Å). Moreover, the modalities of action of quercetin and EGFR were shown in Figure 7J and Figure 9J. Quercetin binds to an AKT1 pocket, composed of Gly796, Ala743, Leu792, Val726, Leu844, Leu718, and Cys797. Three hydrogen bonds, quercetin_O5_: Asp800_OD2_ (2.9 Å), quercetin_O7_: Met793_N_ (3.1 Å) and Gln791_O_ (2.7 Å). Therefore, the interactions between the ligand and the EGFR protein are enhanced. Figure 7K and Figure 9K indicate that quercetin was predicted to interact with GAPDH via Asp35, Phe37, Arg80, Val101, Phe102, Thr99, Pro36, Gly12 and formed two H-bonds with the residues Asn9 (3.1 Å) and Asn34 (3.1 Å). Quercetin could bind to TNF by forming hydrophobic interactions with the neighboring residues Asn65, Leu71, Phe60, Ala62, Leu67 and a hydrogen bond with Glu64 (2.8 Å) (Figures 7L,9L).

**FIGURE 8:**
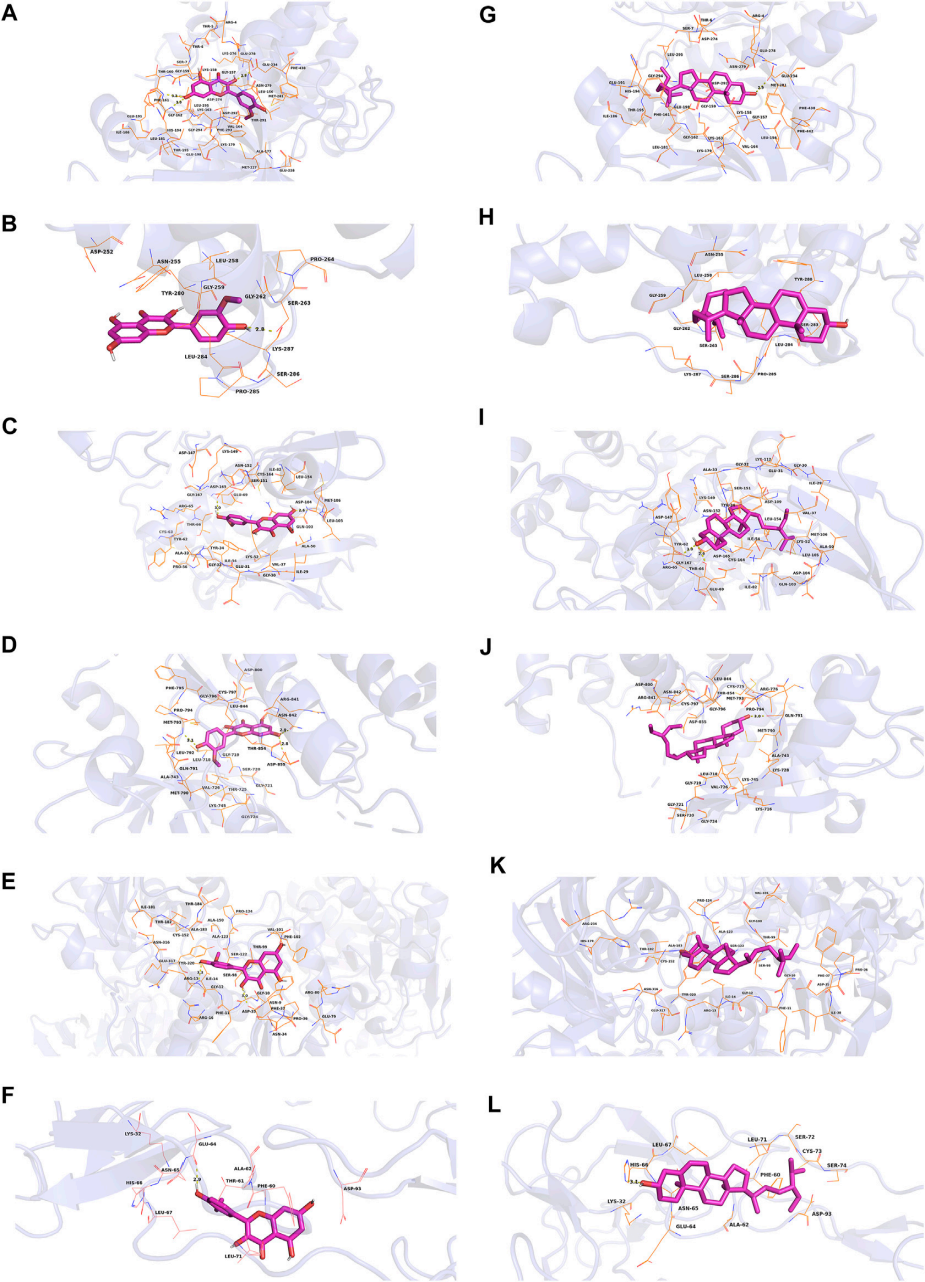
Molecular models of isorhamnetin and beta-sitosterol that bind to the predicted protein targets, shown as 3D diagrams. **(A)** isorhamnetin-AKT1, **(B)** isorhamnetin-MAPK3, **(C)** isorhamnetin-MAPK1, **(D)** isorhamnetin-EGFR, **(E)** isorhamnetin-GAPDH, **(F)** isorhamnetin-TNF, **(G)** beta-sitosterol-AKT1, **(H)** beta-sitosterol-MAPK3, **(I)** beta-sitosterol-MAPK1, **(J)** beta-sitosterol-EGFR, **(K)** beta-sitosterol-GAPDH, (L) beta-sitosterol-TNF.

**FIGURE 9:**
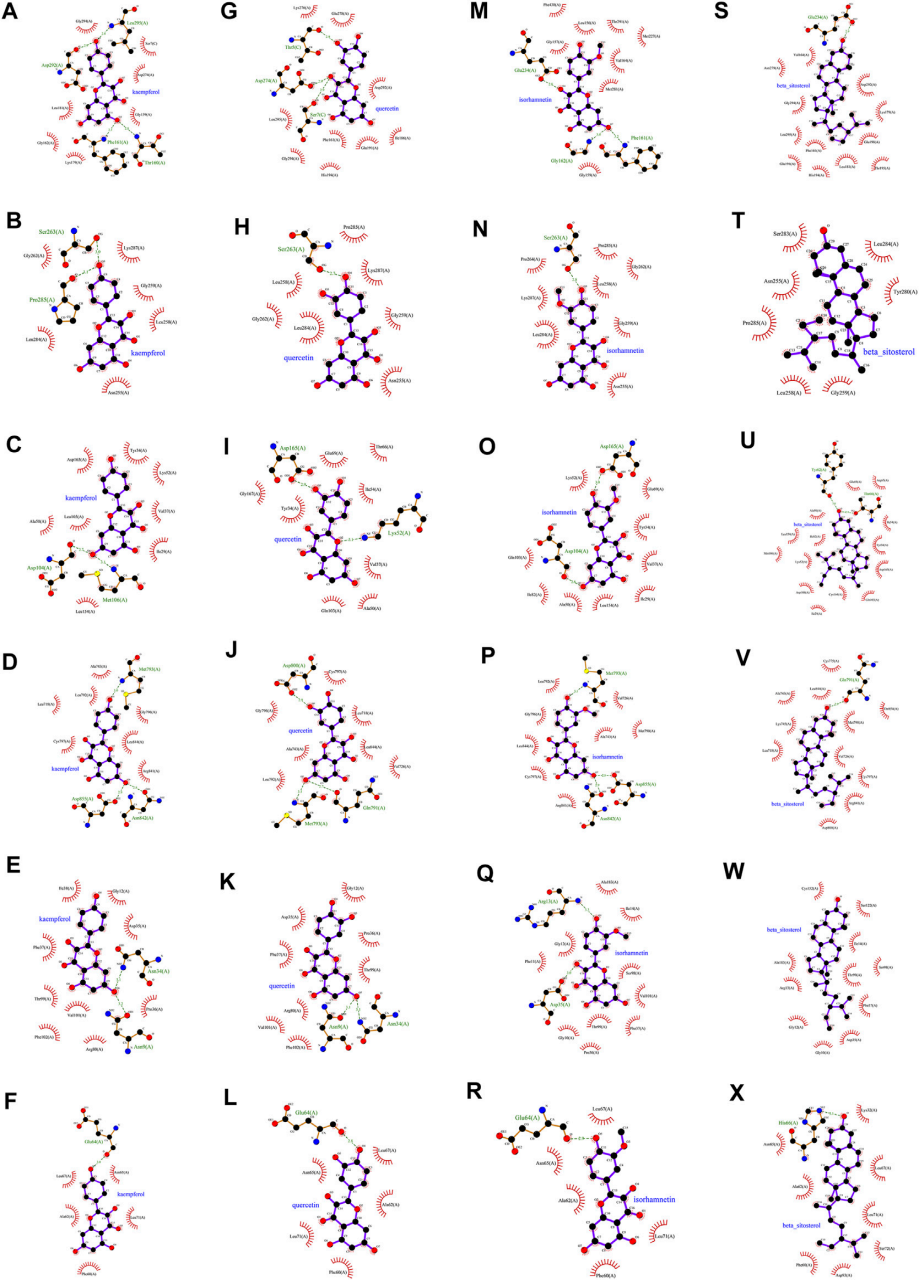
Molecular models of kaempferol, quercetin, isorhamnetin and beta-sitosterol that bind to the predicted protein targets, shown as 2D diagrams. **(A)** Kaempferol-AKT1, **(B)** kaempferol-MAPK3, **(C)** kaempferol-MAPK1, **(D)** kaempferol-EGFR, **(E)** kaempferol-GAPDH, **(F)** kaempferol-TNF, **(G)** quercetin-AKT1, **(H)** quercetin-MAPK3, **(I)** quercetin-MAPK1, **(J)** quercetin-EGFR, **(K)** quercetin-GAPDH, **(L)** quercetin-TNF. **(M)** isorhamnetin-AKT1, **(N)** isorhamnetin-MAPK3, **(O)** isorhamnetin-MAPK1, **(P)** isorhamnetin-EGFR, **(Q)** isorhamnetin-GAPDH, **(R)** isorhamnetin-TNF, **(S)** beta-sitosterol-AKT1, **(T)** beta-sitosterol-MAPK3, **(U)** beta-sitosterol-MAPK1, **(V)** beta-sitosterol-EGFR, **(W)** beta-sitosterol-GAPDH, **(X)** beta-sitosterol-TNF.

According to Figure 8A and Figure 9M, nineteen residues in AKT1 were shown to have hydrophobic interactions with isorhamnetin (Phe438, Gly157, Leu156, Thr291, Met227, Val164, Met281, and Gly159) and three hydrogen bonds [isorhamnetin_O4_: Glu234_OE2_ (2.8 Å), isorhamnetin_O7_: Gly162_N_ (3.0 Å) and Phe161_N_ (3.2 Å)]. As shown in Figure 8B and Figure 9N, isorhamnetin was observed to connect with MAPK3 via Pro264, Lys287, Leu284, Asn255, Gly259, Leu258, Gly262 and Pro285 and form an H-bond with the residue Ser263 (2.8 Å). Figure 8C and Figure 9O showed that isorhamnetin interacts with MAPK1 through hydrophobic interactions with adjacent residues (Lys52, Gln103, Ile82, Ala50, Leu154, Ile29, Val37, Tyr34, and Glu69) and two hydrogen bonds [Asp165_OD2_ (2.8 Å), Asp104_O_ (2.6 Å)]. Moreover, isorhamnetin could interact with EGFR via Leu792, Gly796, Leu844, Cys797, Arg841, Ala743, Met790, and Val726 and formed three hydrogen bonds with the residues Met793_N_ (3.1 Å), Asn842_OD1_ (2.9 Å), Asp855_OD2_ (2.8 Å) (Figures 8D,9P). Besides, isorhamnetin formed hydrophobic interactions with thirteen GAPDH residues (Gly12, Phe11, Gly10, Pro36, Thr99, Phe37, Val101, Ser98, Ile14, and Ala183), and formed two hydrogen bonds with the residues Arg13 (3.3 Å) and Asp35 (3.0 Å) (Figures 8E,9Q). Isorhamnetin also interacted with TNF via Asn65, Ala62, Phe60, Leu71, Leu67 and formed an H-bond with the residue Glu64 (2.9 Å) (Figures 8F,9R).

The action modes of beta-sitosterol and AKT1 are shown in Figure 8G and Figure 9S. Beta-sitosterol bound to a pocket in AKT1, composing of Val164, Asn279, Gly294, Leu295, Phe161, Glu191, His194, Leu181, Thr195, Glu198, Lys179, Asp292 and a hydrogen bond, Glu234 (2.9 Å). As shown in Figure 8H and Figure 9T, it was predicted that beta-sitosterol could interact with MAPK3 through Ser283, Asn255, Pro285, Leu285, Gly259, Tyr280, Leu284. According to the analysis results shown in Figure 8I and Figure 9U, beta-sitosterol was observed to form hydrophobic interactions with 14 residues in MAPK1 (Ala50, Leu154, Ile82, Met106, Lys52, Asp104, Ile29, Cys164, Gln103, Asp165, Tyr34, Ile54, Arg65, and Glu69) and 2 H-bond with the residue Tyr62 (3.0 Å) and Thr66 (2.9 Å). Beta-sitosterol could bind to the EGFR via hydrophobic interactions with adjacent residues Cys775, Leu844, Ala743, Lys745, Leu718, Asp800, Arg841, Cys797, Val726, Met790, Thr854 and an H-bond with Gln791 (2.9 Å) (Figures 8J,9V). Moreover, beta-sitosterol was shown to bind to GAPDH via hydrophobic interactions with neighboring residues Cys152, Ala183, Arg13, Gly12, Gly10, Asp35, Phe37, Thr99, Ser98, Ile14, Ser122 (Figures 8K,9W). Beta-sitosterol also interacted with TNF *via* Asn65, Ala62, Phe60, Asp93, Ser72, Leu71, Leu67, Lys32 and a hydrogen bond with His66 (3.1 Å) (Figures 8L,9X).

### 3.4 UHPLC-Q-orbitrap HRMS

**FIGURE 10:**
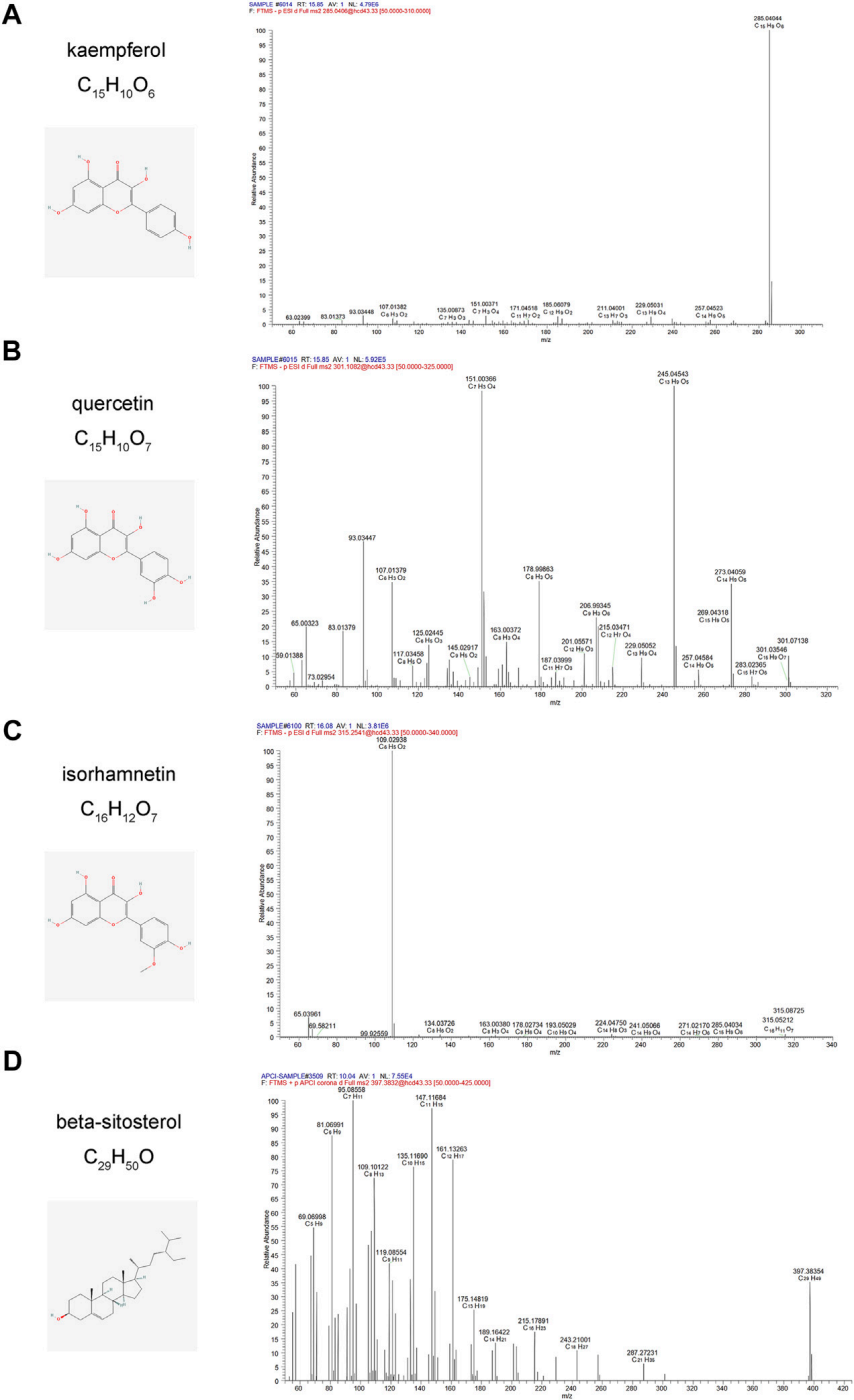
Mass spectrum of kaempferol, quercetin, isorhamnetin, and beta-sitosterol from CCMM extract in the negative and positive ion modes, respectively. **(A)** ESI-MS/MS spectra of kaempferol from CCMM extract. **(B)** ESI-MS/MS spectra of quercetin from CCMM extract. **(C)** ESI-MS/MS spectra of isorhamnetin from CCMM extract. **(D)** APCI-MS/MS spectra of beta-sitosterol from CCMM extract.

The active components of CCMM were analyzed using a Q-Exactive orbitrap mass spectrometer. We determined the element compositions of kaempferol, quercetin, isorhamnetin, beta-sitosterol, and MS^2^ fragment ions using their precise mass measurements. Figure 10 illustrates the retention periods, molecular formulae, and high-resolution MS^2^ fragment ions in negative and positive modes. As illustrated in Figure 10, Compound Discover and Xcalibur software were used to identify kaempferol, quercetin, isorhamnetin, and beta-sitosterol, which corroborated the network pharmacology results. In addition, the MS^2^ spectrum of kaempferol, quercetin, isorhamnetin, beta-sitosterol standards are shown in Supplementary Figure S16. Jaranol, (6aR,11aR)-9,10-dimethoxy-6a, 11a-dihydro-6H-benzofurano [3,2-c]chromen-3-ol, isoflavanone, 1-hydroxy-3-methoxy-9,10-anthraquinone, 3,9-di-O-methylnissolin, isorhamnetin, morin, (3R)-3-(2-hydroxy-3,4-dimethoxyphenyl) chroman-7-ol, cnidilin, sitosterol alpha1, and NSC63551 have been identified in CCMM extract. These related MS^2^ spectrum are shown in Supplementary Figures S17, S18.

### 3.5 Experimental validation

#### 3.5.1 The sperm quality of OA mice in the treatment of kaempferol, quercetin, isorhamnetin, beta-sitosterol and Chinese materia medica

**FIGURE 11:**
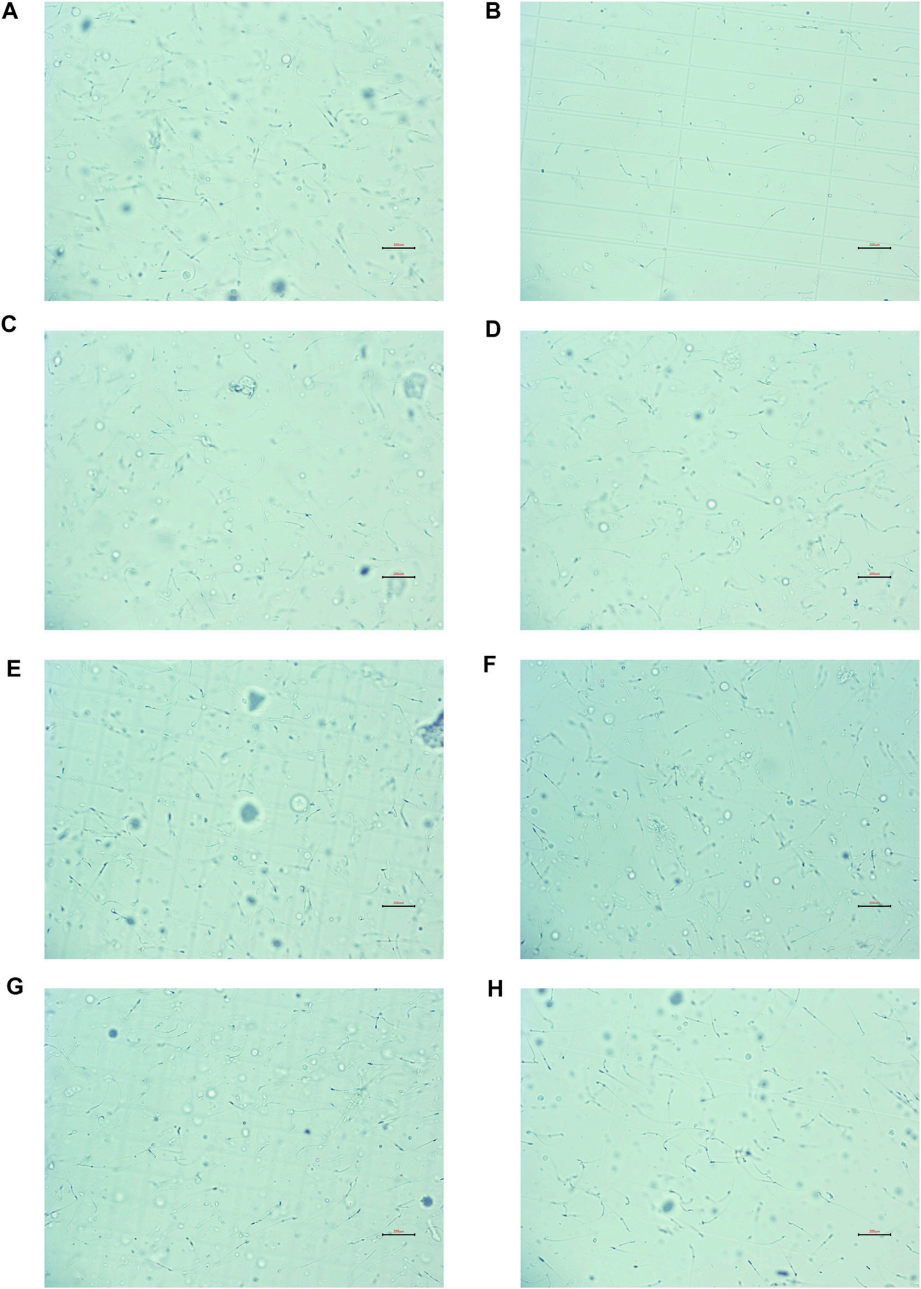
The sperm morphology (×200). **(A)** The normal control group. **(B)** The model control group. **(C)** The kaempferol group. **(D)** The quercetin group. **(E)** The isorhamnetin group. **(F)** The beta-sitosterol group. **(G)** The low-dose CCMM group. **(H)** The high-dose CCMM group. Data: n = 8, experiments performed in triplicate.

**FIGURE 12:**
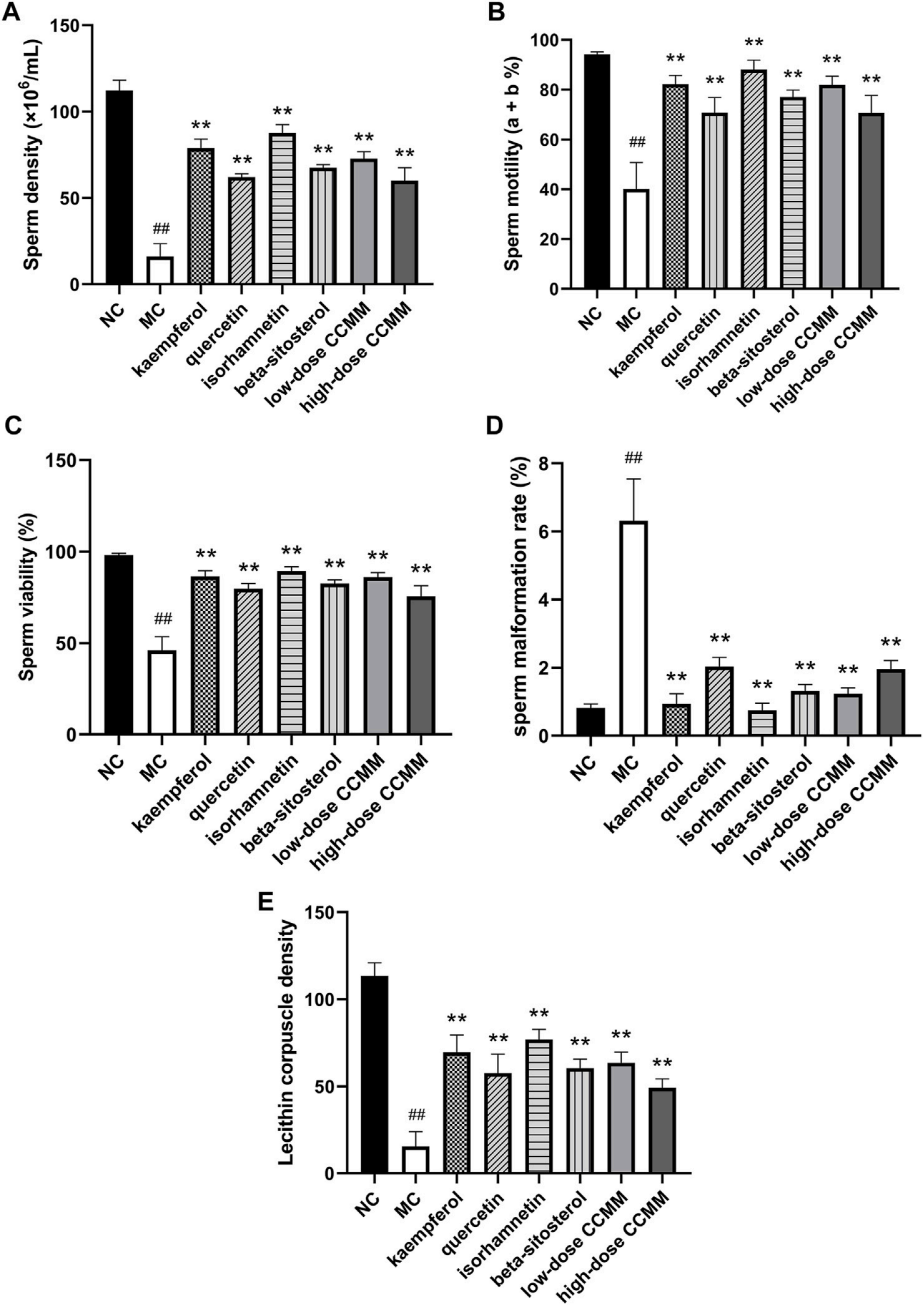
The sperm quality of each group. **(A)** The normal control group. **(B)** The model control group. **(C)** The kaempferol group. **(D)** The quercetin group. **(E)** The isorhamnetin group. **(F)** The beta-sitosterol group. **(G)** The low-dose CCMM group. **(H)** The high-dose CCMM group. Data: n = 8, mean ± SD, experiments performed in triplicate. ^##^ *p* < 0.01 versus the NC group, ^**^ *p* < 0.01 versus the MC group.

In contrast to the NC group, CP dramatically lowered the density, motility, and viability of sperm, and Lecithin corpuscle density in the MC group, while increasing the sperm malformation rate (all *p* < 0.01). This demonstrated that the OA mouse model had been constructed efficiently (all *p* < 0.01). Compared to the MC group, the kaempferol, quercetin, isorhamnetin, beta-sitosterol, and low- and high-dose CCMM groups enhanced the density, motility, viability of sperm, and lecithin corpuscle density, while reducing the rate of sperm malformation (all *p* < 0.01). Figure 11 represents the sperm morphology. The results are presented in Figure 12.

#### 3.5.2 Histopathological analysis of OA mice in the treatment of kaempferol, quercetin, isorhamnetin, beta-sitosterol and Chinese materia medica

**FIGURE 13:**
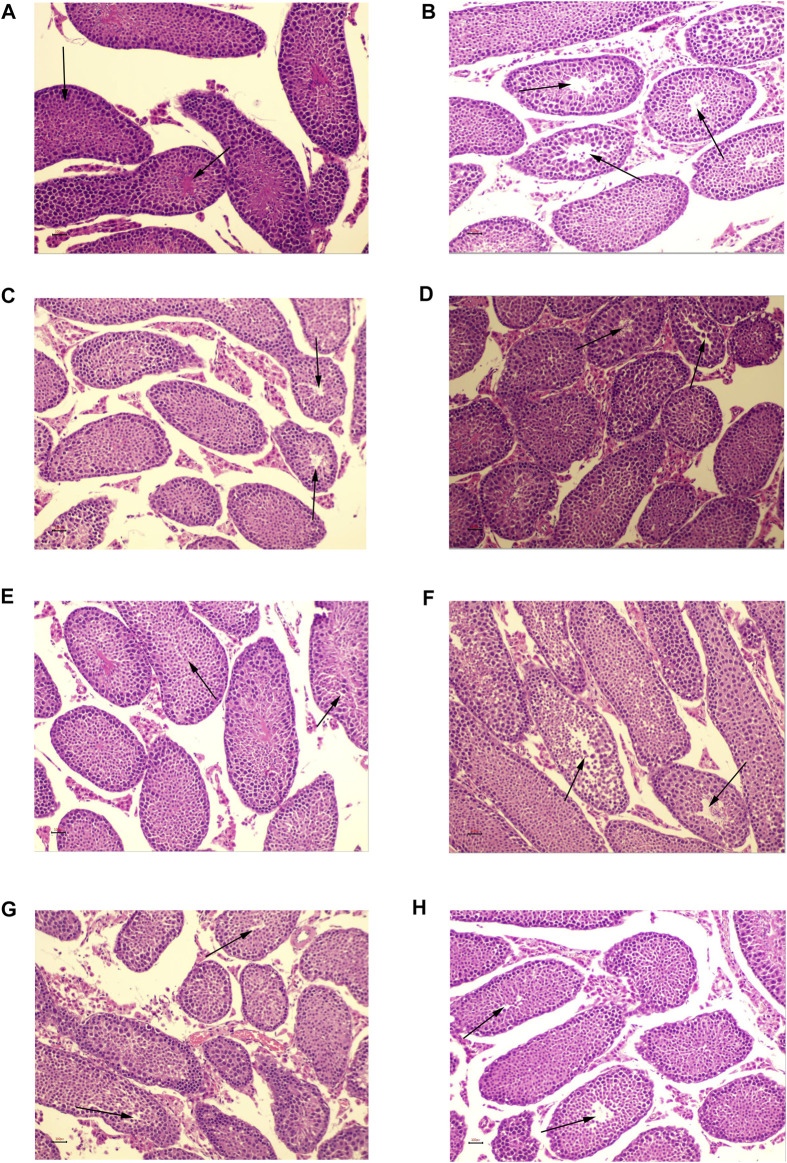
HE staining of testicular tissues (×200). **(A)** The normal control group. **(B)** The model control group. **(C)** The kaempferol group. **(D)** The quercetin group. **(E)** The isorhamnetin group. **(F)** The beta-sitosterol group. **(G)** The low-dose CCMM group. **(H)** The high-dose CCMM group. Data: n = 5, experiments performed in triplicate.

In the NC group, there were no evident pathological abnormalities in the quantity or shape of seminiferous tubules. The tracheal seminiferous tubules were firmly packed, and the basement membrane-interstitium border was clean and flat. At all levels of the seminiferous tubules, the spermatogenic cells were neatly and orderly organized (Figure 13A). The testicular tissue structures of the MC group were grossly normal. The number of deformed and collapsed germinal tubules increased, and the germinal cells in the germinal tubules were shed and disorganized. The spermatogenic cells at all levels were reduced. Thinning of the spermatogenic epithelium, shortage of sperm bundles, loosely arranged Leydig cells, and partial hyaline degeneration of interstitial tissue was shown in Figure 13B. In the kaempferol, quercetin, isorhamnetin, beta-sitosterol, and low- and high-dose CCMM groups, the testicular tissue structures were normal. The lumen of the seminiferous tubule was regular in testicular tissue. In the seminiferous tubules, the spermatogenic cells were regularly arranged, and the structure was tight. All the different stages of spermatogenic cells are present within the seminiferous tubules. The abnormal vacuoles of seminiferous tubules were present only occasionally. The epididymal lumen contains mature spermatozoa. The shedding cells can be found in some of the seminiferous tubules. Some of the interstitial spaces showed varying degrees of enlargement. A fraction of the thickened basement membrane was observed occasionally (Figures 13C–H).

#### 3.5.3 Western blot analysis of OA mice in the treatment of kaempferol, quercetin, isorhamnetin, beta-sitosterol and Chinese materia medica

**FIGURE 14:**
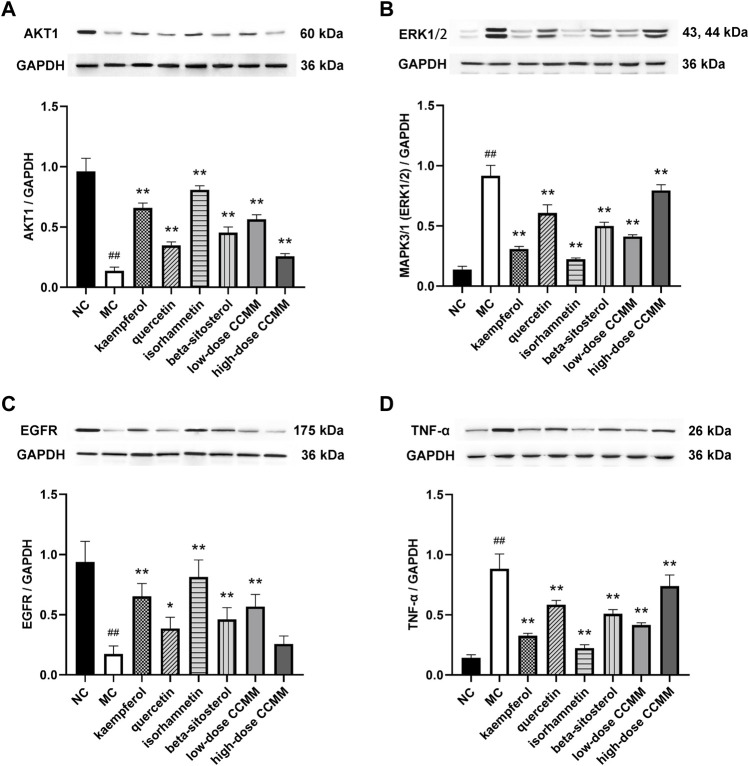
Western blot analysis and quantitative data of AKT1 **(A)**, MAPK3/1 (ERK1/2) **(B)**, EGFR **(C)**, and TNF-α **(D)** of testicular tissues in each group. Data: n = 5, mean ± SD, experiments performed in triplicate. ^##^ *p* < 0.01 versus the NC group, ^*^ *p* < 0.05 versus the MC group, ^**^ *p* < 0.01 versus the MC group.

Western blot and quantitative data show that CP significantly decreased the protein expressions of AKT1 and EGFR, while increasing the protein expressions of MAPK3/1 (ERK1/2) and TNF-α, compared to the NC group (all *p* < 0.01). In comparison to the MC group, the kaempferol, quercetin, isorhamnetin, beta-sitosterol, and CCMM enhanced the protein expressions of AKT1 (*p* < 0.01) and EGFR (*p* < 0.05), while reducing the protein expressions of MAPK3/1 (ERK1/2) (*p* < 0.01) and TNF-α (*p* < 0.01) (Figure 14).

## 4 Discussion

Infertility affects approximately 15% of reproductive-aged couples globally, of which MI accounts for approximately 50% of instances (Sharlip et al., 2002). However, the pathogenesis and mechanism of MI remain under investigation (Choy and Eisenberg, 2018). Currently, surgical interventions and pharmacological therapies for MI each have their limitations (Duca et al., 2019). Thus, it is urgent for MI management to identify novel therapeutic drugs or to develop effective treatment strategies. For thousands of years, TCM has been utilized to treat MI, but its components, mechanisms, and functions remain ambiguous, limiting its therapeutic applicability. In order to solve this problem, we adopted an integrated approach including data mining, network pharmacology, molecular docking, UHPLC-Q-Orbitrap HRMS, and experimental validation.

**FIGURE 15:**
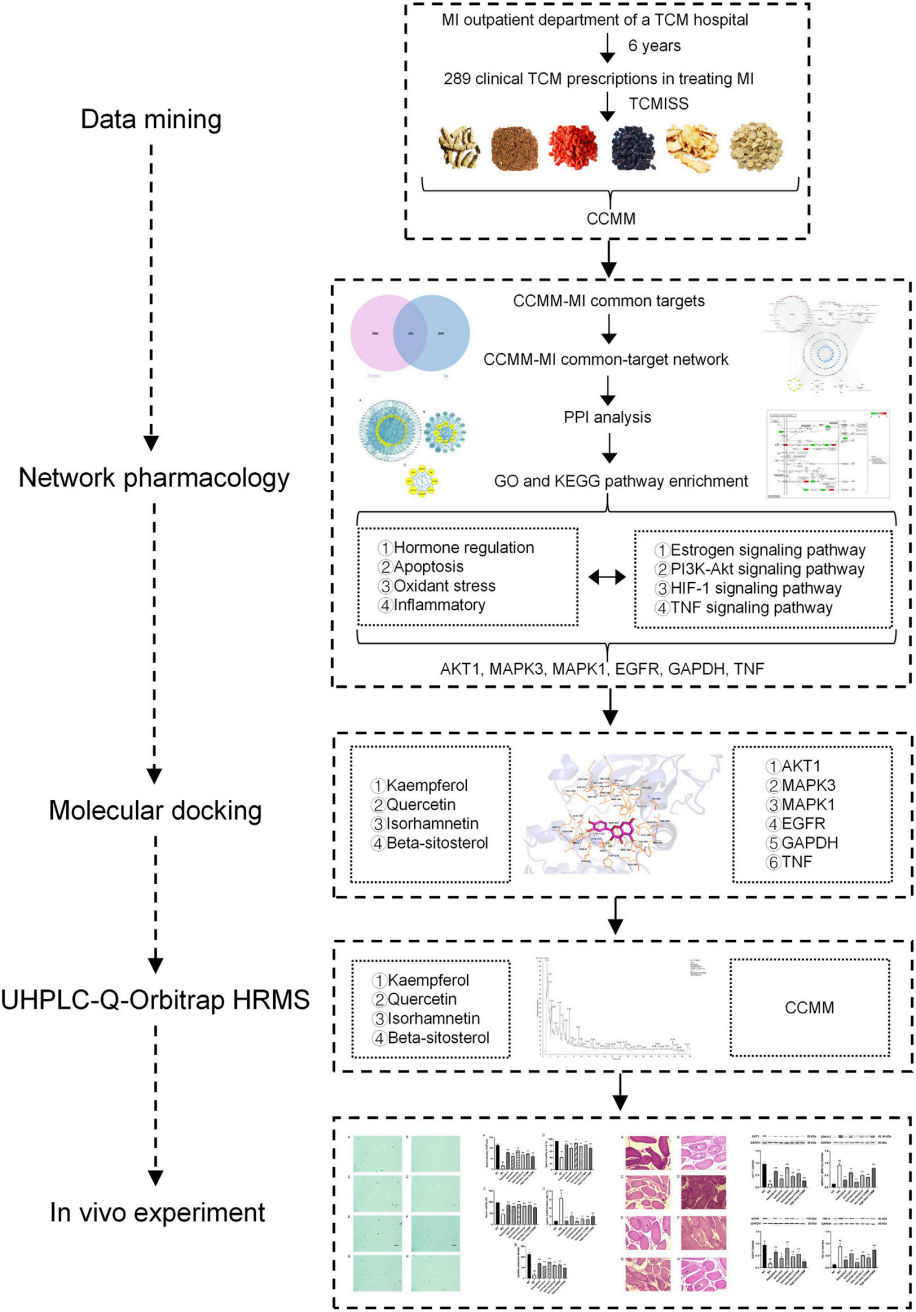
The experimental flow of this study. MI, male infertility; TCM, traditional Chinese medicine; TCMISS, TCM Inheritance Support System; CCMM, Core Chinese Materia Medica; PPI, protein-protein interaction; GO, Gene Ontology; KEGG, Kyoto Encyclopedia of Genes and Genomes.

By data mining, a total of 289 clinical TCM prescriptions were collected from the outpatient department of a TCM hospital. CCMM were screened by TCMISS, which were Gynochthodes officinalis (F.C.How) Razafim. and B. Bremer, Cuscuta chinensis Lam., Lycium barbarum L., Morus alba L., Angelica sinensis (Oliv.) Diels, Corethrodendron multijugum (Maxim.) B.H.Choi and H. Ohashi By using the network pharmacology methods, 98 components and 816 targets of CCMM, and 671 MI-related targets were obtained from 10 databases. 127 common targets between CCMM and MI were obtained by the Venn diagram. The results of network pharmacology showed that kaempferol, quercetin, isorhamnetin, and beta-sitosterol are four core components. AKT1, MAPK3, MAPK1, EGFR, GAPDH, and TNF are six key targets. Estrogen signaling pathway, PI3K-Akt signaling pathway, HIF-1 signaling pathway, and TNF signaling pathway are four vital signaling pathways of CCMM of clinical TCM prescriptions on MI. The key targets and vital signaling pathways of CCMM for treating MI are mainly related to hormone regulation, anti-apoptosis, anti-oxidant stress, and anti-inflammatory. Based on the molecular docking strategy, we verified the strong interactions between four core components and six key targets. By UHPLC-Q-Orbitrap HRMS analysis, four core components of CCMM were successfully identified. In a mouse model of MI, we found that CCMM and four core components could improve the density, motility, viability of sperm, lecithin corpuscle density, decrease the rate of sperm malformation and testis tissue damage, and regulate the proein expressions of AKT1, MAPK3/1, EGFR, and TNF-α. It showed that the results of data mining, network pharmacology, and molecular docking were further validated by UHPLC-Q-Orbitrap HRMS analysis and *in vivo* experiments (Figure 15).

In detail, kaempferol could protect sperm from estrogen-induced oxidative DD (Anderson et al., 2003). Kaempferol restored the motility of aluminum-exposed human sperm cells and reduced the generation of malondialdehyde (MDA), a lipid peroxidation marker, in an *in vitro* research (Jamalan et al., 2016). Indirectly, quercetin has been shown to stimulate sex organs at both the cell and organ levels (Taepongsorat et al., 2008), and shows outstanding beneficial effects on the serum total testosterone (Khaki et al., 2010). Isorhamnetin is a kind of flavonoid and a direct metabolite of quercetin. Isorhamnetin was maintained longer than quercetin in plasma (Lee et al., 2008). It possesses antioxidant and anti-inflammatory properties (Boesch-Saadatmandi et al., 2011; Dong et al., 2014). Beta-sitosterol is a naturally occurring phytosterol with a steroidal moiety that has the ability to prevent tumor development, alter immunological response, and act as an antioxidant. Beta-sitosterol is being investigated as a possible chemopreventive drug for the treatment of a number of cancers, including prostate and breast cancer (Patel et al., 2017).

AKT1 is thought to regulate cell growth, survival, metabolism, and proliferation (Poplinski et al., 2010). Additionally, AKT1 inhibits radiation-induced death of germ cells *in vivo* (Rasoulpour et al., 2006) and increases thyroid hormone’s effects on postnatal testis growth (Santos-Ahmed et al., 2011). MAPKs have been associated with abnormal spermatogenesis and germ cell and Sertoli cell dysfunction, leading to reduced sperm quality and male reproductive problems (Li et al., 2009). MAPK3 and MAPK1 are required for cell cycle progression and apoptosis in humans (Cocchia et al., 2011). The EGFR is partly stimulated during the capacitation process by protein kinase A (PKA), leading to the activation of phospholipase D (PLD) and actin polymerization (Breitbart and Etkovitz, 2011). GAPDH is particularly important in the testis for spermatogenesis and decreased sperm motility caused by male infertility (Gunnarsson et al., 2007). TNF-α, a multifunctional cytokine, is involved in a variety of critical processes including cell survival, proliferation, differentiation, inflammation, germ cell death, and spermatogenesis regulation (Wang and Lin, 2008; Bami et al., 2017).

Among the signaling pathways identified, the estrogen signaling pathway is the most significant. Estrogens have a role in the pathophysiology of male infertility associated with varicocele (Guido et al., 2011). Estrogen stimulation has been shown to directly affect germ cell apoptosis and to alter the communication among germ cells, consequently affecting their apoptosis (Alves, 2013), which might have a profound effect on MI. Abnormal activation of the PI3K-Akt signaling pathway may contribute to the spread of prostate cancer cells and the disease’s development (Shukla et al., 2007). The hypoxia-inducible factor (HIF)-1 protein is required for the human body to respond appropriately to low oxygen levels or hypoxia (Velickovic and Stefanovic, 2014). TNF family is regarded to stimulate NF-κB, thus implicating in varicocele-mediated pathogenesis (Celik et al., 2013).

CP is a novel chemotherapeutic agent that decreases fertility in people treated with it (Haque et al., 2001; Das et al., 2002; Ghosh et al., 2002). The tissue and epididymis may be adversely affected by CP, according to previous investigations (Trasler et al., 1988). Additionally, it may decrease sperm production in the testes and sperm maturation in the epididymis (Trasler et al., 1986). Patients treated with CP for at least four months have developed a variety of MI complications (Qureshi et al., 1972). CP was the most detrimental to the testis (Howell and Shalet, 2005). The use of CP as an alkylating agent and cytotoxic agent has a considerable impact on sperm consistency and fertility (Goldberg et al., 1986). Male animals’ sperm characteristics may be affected by CP therapy (Aitken and Clarkson, 1987). Kaempferol, quercetin isorhamnetin, beta-sitosterol, and CCMM improved sperm quality while decreasing testis tissue damage in the MI mouse model caused by CP, confirming the findings of data mining, network pharmacology, and molecular docking.

According to the network pharmacology evaluation method guidance (Li, 2021), our research also has some shortcomings. The specific interactions between all the drug components, proteins and multiple signal pathways involved in it need to be further studied. The comprehensive approach integrated data mining, network pharmacology, molecular docking, UHPLC-Q-Orbitrap HRMS, and experimental validation might be a powerful new way to elucidate the mystery of TCM.

## 5 Conclusion

In summary, based on the comprehensive approach integrated data mining, network pharmacology, molecular docking, UHPLC-Q-Orbitrap HRMS, and experimental validation, we found that Gynochthodes officinalis (F.C.How) Razafim. and B. Bremer, Cuscuta chinensis Lam., Lycium barbarum L., Morus alba L., Angelica sinensis (Oliv.) Diels, Corethrodendron multijugum (Maxim.) B.H.Choi and H. Ohashi are CCMM of clinical TCM prescriptions for treating MI. The core components are kaempferol, quercetin, isorhamnetin, and beta-sitosterol. The mechanism and functions of CCMM for treating MI are hormone regulation, anti-apoptosis, anti-oxidant stress, and anti-inflammatory. Molecular docking was used to confirm that four core components and six key targets had strong interactions. UHPLC-Q-Orbitrap HRMS analysis was used to recognize the core components in CCMM extract. Finally, *in vivo* experiments proved that CCMM and the core components could improve the density, motility, viability of sperm, lecithin corpuscle density, decrease the rate of sperm malformation and testis tissue damage, and regulate the protein expressions of AKT1, MAPK3/1, EGFR, TNF-α in the MI mice. This study not only illustrated the components, mechanisms, and functions of clinical TCM prescriptions for MI, but also elaborated on TCM’s multi-component, multi-target, and multi-pathway characteristics in disease treatment.

## Data Availability

The original contributions presented in the study are included in the article/Supplementary Material, further inquiries can be directed to the corresponding authors.
