# Supplementary material for: An integrative approach to uncover the components, mechanisms, and functions of traditional Chinese medicine prescriptions on male infertility
Source: Front Pharmacol. 2022 Aug 11;13:794448. doi: 10.3389/fphar.2022.794448 (PMC9403420; doi:10.3389/fphar.2022.794448)
Supplement: Supplementary file 10 [file DataSheet11.docx]

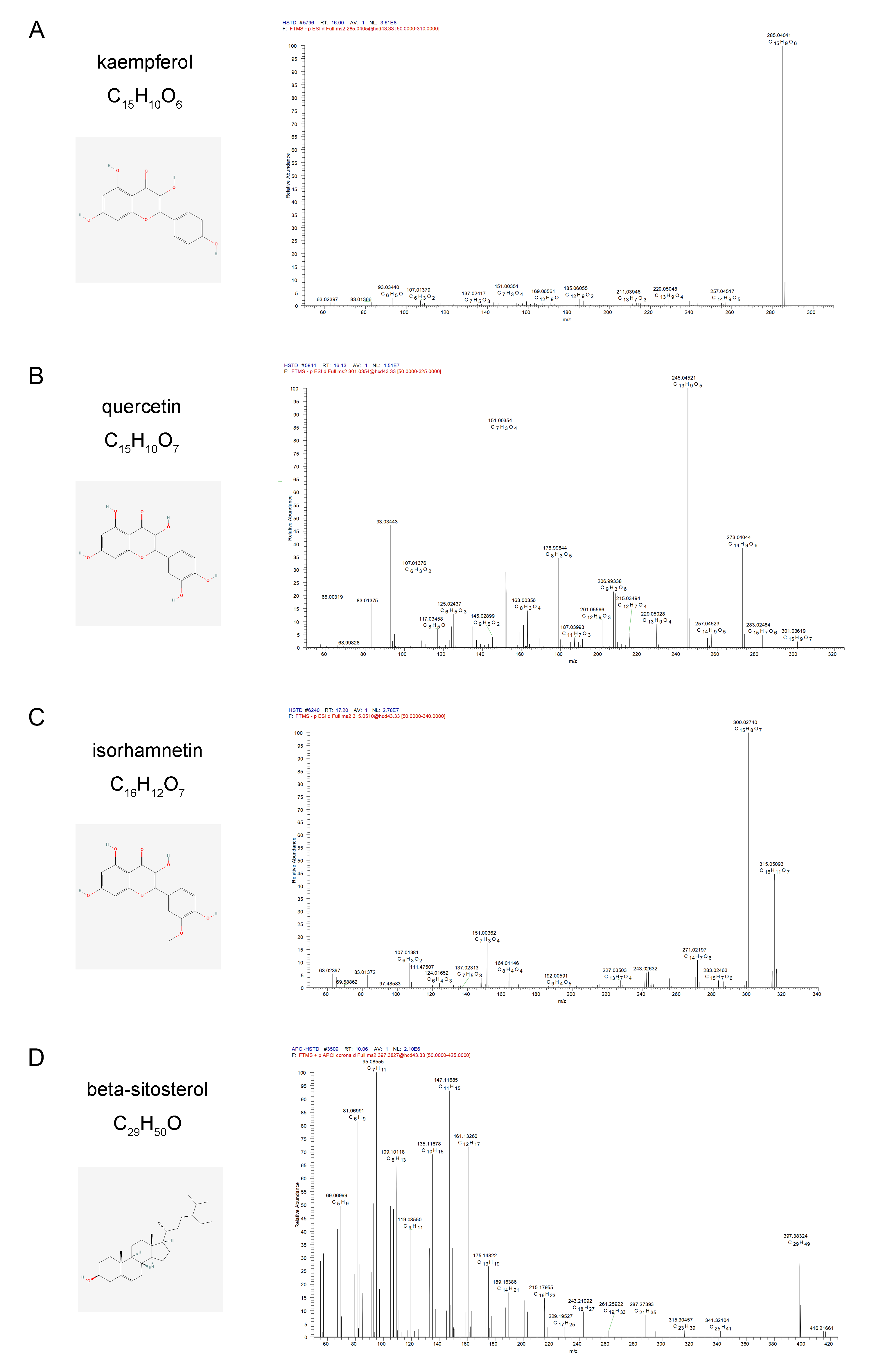


**FIGURE 16**: Mass spectrum of kaempferol, quercetin, isorhamnetin, and beta-sitosterol standards in the negative and positive ion modes, respectively. (A) ESI-MS/MS spectra of kaempferol standard. (B) ESI-MS/MS spectra of quercetin standard. (C) ESI-MS/MS spectra of isorhamnetin standard. (D) APCI-MS/MS spectra of beta-sitosterol standard.


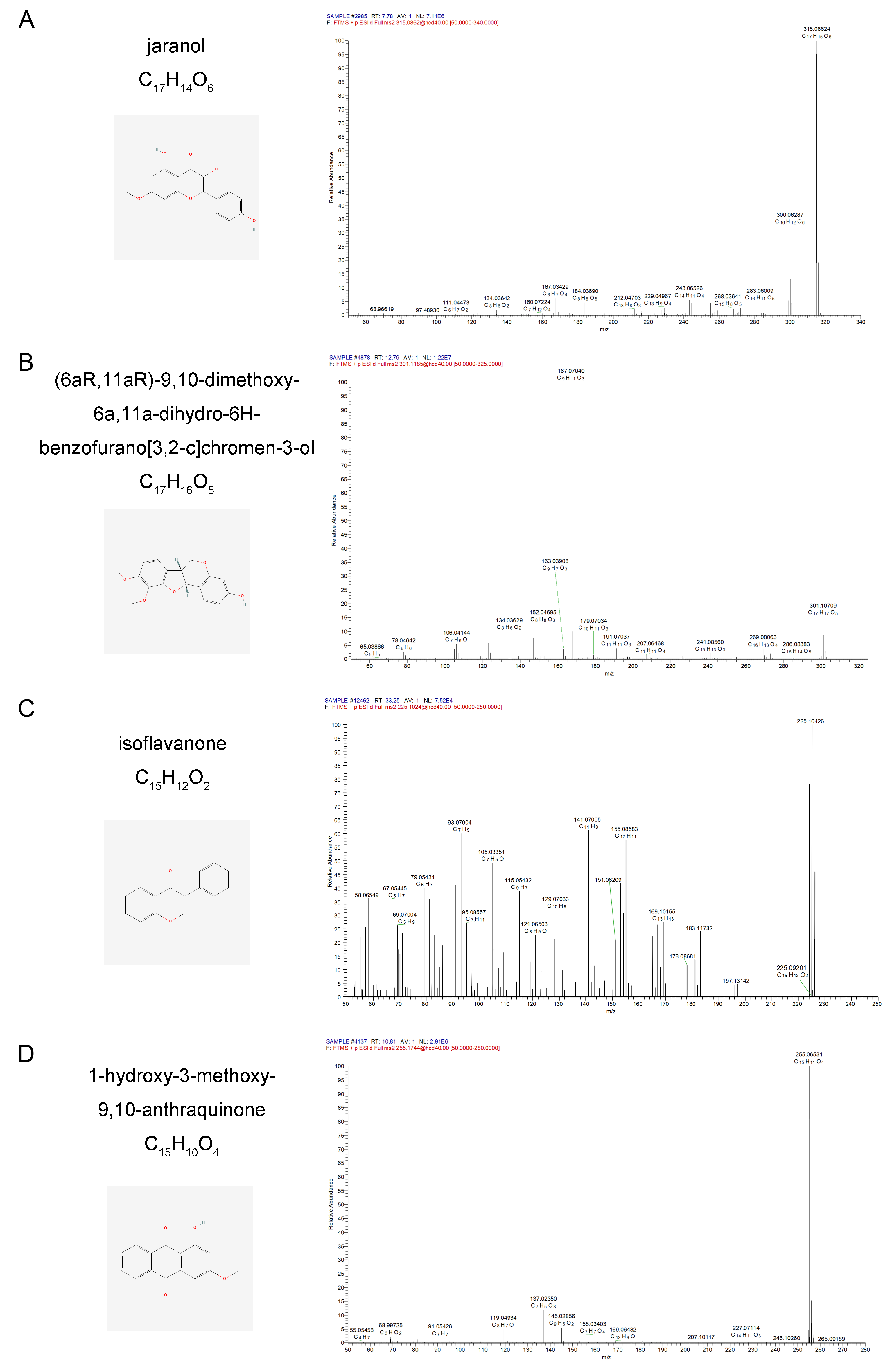


**FIGURE 17**: Mass spectrum of jaranol, (6aR,11aR)-9,10-dimethoxy-6a,11a-dihydro-6H-benzofurano[3,2-c]chromen-3-ol, isoflavanone, and 1-hydroxy-3-methoxy-9,10-anthraquinone from CCMM extract in the negative and positive ion modes, respectively. (A) ESI-MS/MS spectra of jaranol from CCMM extract. (B) ESI-MS/MS spectra of (6aR,11aR)-9,10-dimethoxy-6a,11a-dihydro-6H-benzofurano[3,2-c]chromen-3-ol from CCMM extract. (C) ESI-MS/MS spectra of isoflavanone from CCMM extract. (D) ESI-MS/MS spectra of 1-hydroxy-3-methoxy-9,10-anthraquinone from CCMM extract.


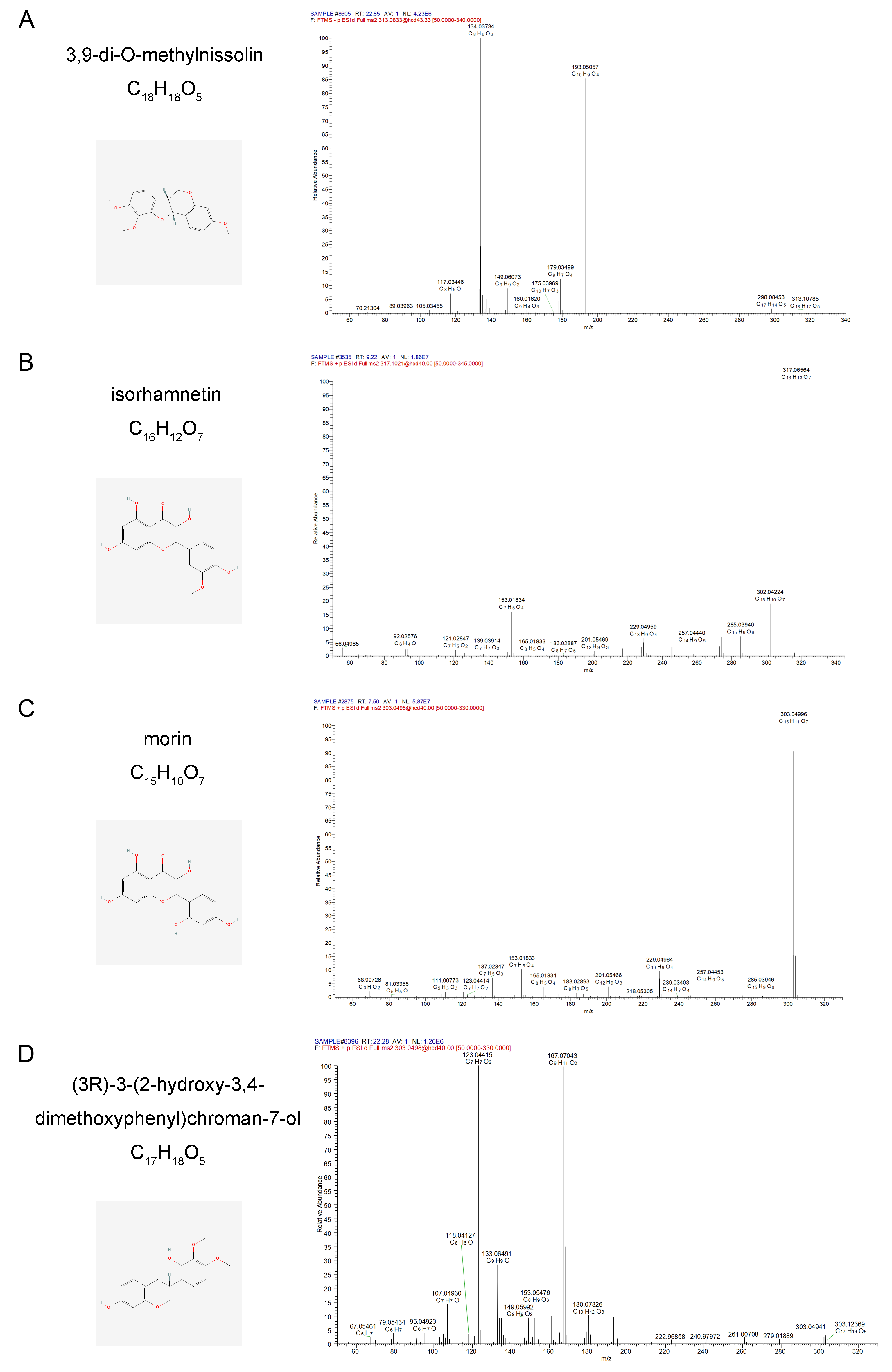


**FIGURE 18**: Mass spectrum of 3,9-di-O-methylnissolin, isorhamnetin, morin, and (3R)-3-(2-hydroxy-3,4-dimethoxyphenyl)chroman-7-ol from CCMM extract in the negative and positive ion modes, respectively. (A) ESI-MS/MS spectra of 3,9-di-O-methylnissolin from CCMM extract. (B) ESI-MS/MS spectra of isorhamnetin from CCMM extract. (C) ESI-MS/MS spectra of morin from CCMM extract. (D) ESI-MS/MS spectra of (3R)-3-(2-hydroxy-3,4-dimethoxyphenyl)chroman-7-ol from CCMM extract.


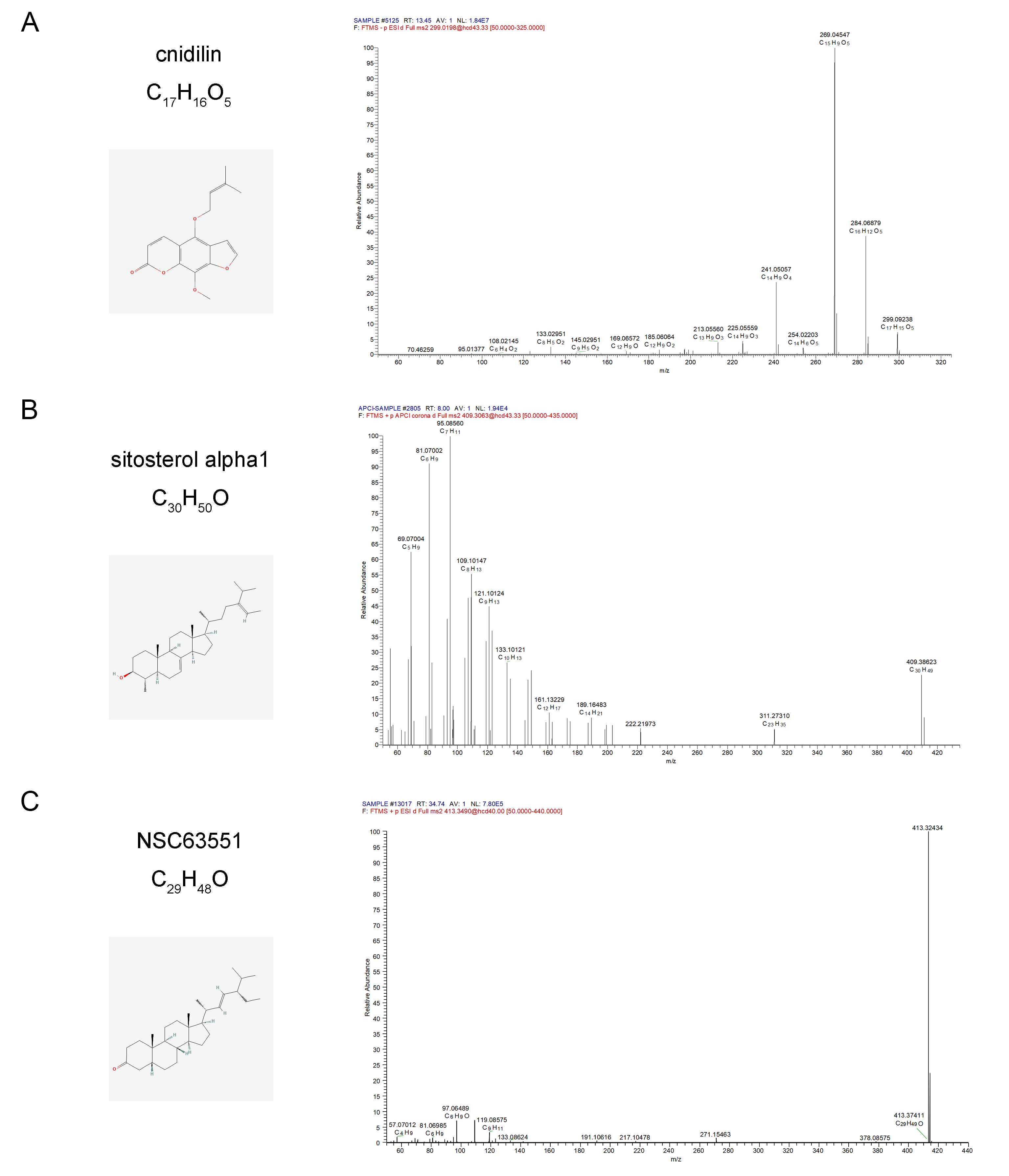


**FIGURE 19**: Mass spectrum of cnidilin, sitosterol alpha1, and NSC63551 from CCMM extract in the negative and positive ion modes, respectively. (A) ESI-MS/MS spectra of cnidilin from CCMM extract. (B) APCI-MS/MS spectra of sitosterol alpha1 from CCMM extract. (C) ESI-MS/MS spectra of NSC63551 from CCMM extract.
